# Supplementary material for: The core PCP protein Prickle2 regulates axon number and AIS maturation by binding to AnkG and modulating microtubule bundling
Source: Sci Adv. 2022 Sep 9;8(36):eabo6333. doi: 10.1126/sciadv.abo6333 (PMC9462691; doi:10.1126/sciadv.abo6333)
Supplement: 20220909-1 [file sciadv.abo6333.v1.pdf]

DEVELOPMENTAL NEUROSCIENCE

Copyright © 2022 The Authors, some rights reserved; exclusive licensee American Association for the Advancement of Science. No claim to original U.S. Government Works. Distributed under a Creative Commons Attribution License 4.0 (CC BY).

The core PCP protein Prickle2 regulates axon number and AIS maturation by binding to AnkG and modulating microtubule bundling

Ana Dorrego-Rivas[1], Jerome Ezan[1], Maïté M. Moreau[1], Sonia Poirault-Chassac[1], Nathalie Aubailly[1], Julie De Neve[1], Camille Blanchard[1], Francis Castets[2], Amélie Fréal[3], Arne Battefeld[4], Nathalie Sans[1]†, Mireille Montcouquiol[1]*†‡

Core planar cell polarity (PCP) genes, which are involved in various neurodevelopmental disorders such as neural tube closure, epilepsy, and autism spectrum disorder, have poorly defined molecular signatures in neurons, mostly synapse-centric. Here, we show that the core PCP protein Prickle-like protein 2 (Prickle2) controls neuronal polarity and is a previously unidentified member of the axonal initial segment (AIS) proteome. We found that Prickle2 is present and colocalizes with AnkG480, the AIS master organizer, in the earliest stages of axonal specification and AIS formation. Furthermore, by binding to and regulating AnkG480, Prickle2 modulates its ability to bundle microtubules, a crucial mechanism for establishing neuronal polarity and AIS formation. Prickle2 depletion alters cytoskeleton organization, and Prickle2 levels determine both axon number and AIS maturation. Last, early Prickle2 depletion produces impaired action potential firing.

INTRODUCTION

During development, most neurons acquire an asymmetric cellular morphology, with two molecularly and functionally distinct compartments, the axon and the somatodendritic domain. This asymmetry determines the polarity of the neuron, initiated at the time of axonal specification. A substructure called the axonal initial segment (AIS) is located at the junction between the axon and somatodendritic compartment. The AIS depends on neuronal polarity for its establishment and then subsequently acts to maintain this polarity in mature neurons (*1–3*). It is also a key regulator of action potential (AP) initiation and therefore neuronal excitability (*4*, *5*). Such a key compartment for neuronal function relies on a robust cytoskeleton network: Microtubules (MTs) at the AIS form highly packed and parallel fascicles, and actin presents in the form of patches and subcortical periodic actin rings that extend along the entire axon (*6–9*). In mature neurons, the axon transmits signals over long distances, while most dendrites receive information via thousands of spines. The absence of axon specification or the disruption of the maintenance of polarity has broad pathological outcomes, including neurodegenerative disorders (*10*, *11*).

Axon formation involves sequential and interconnected molecular cellular events, many of which also participate in the formation and stabilization of the AIS (*12–18*). At the molecular level, neuronal polarity is presumed to share molecular components not only with the better-defined epithelial apicobasal polarity but also with some cytoskeletal dynamic regulators, such as small guanosine triphosphatases and Wnts (*19–22*). Another common trait of both types of polarity is the establishment of a complex but organized cytoskeletal meshwork.

In 2003, we established that planar cell polarity (PCP) genes that are evolutionarily conserved from invertebrates to mammals also define a PCP axis in mammalian epithelia that is dependent on, and perpendicular to, the more classical apicobasal axis (*23*, *24*). If neuronal polarity modules share some similarities with those of epithelial cells, no clear neuronal PCP is defined in neurons or the brain due to the lack of a plane of reference. Nevertheless, mutations in PCP genes are linked to neurodevelopmental and neurological pathologies, including autism, epilepsy, intellectual disability, and neural tube defects (*25*). *Planar Cell Polarity Protein 2 or Prickle 2 (Pk2)* is one of these core PCP genes whose mutation has been linked to autism spectrum disorders (ASDs) and epilepsy (*26*, *27*). Extensive studies have examined the role of Prickle in epithelial PCP (*28–30*), and in neurons, Prickle2 has been implicated in synaptic function (*26*, *27*, *31*, *32*). However, a clear molecular role for the protein is lacking, both in invertebrates and vertebrates (*33*, *34*).

In this study, we show that Prickle2 is one of the earliest molecules present at the AIS with Ankyrin-G (AnkG) and that its expression is maintained in mature neurons. Prickle2 binds to AnkG, and this interaction promotes the generation of MT bundles via the end-binding 1/3 (EB1/3) sites on AnkG. Prickle2 depletion in vitro and in vivo affects axon formation, with a concomitant substantial decrease in the main AIS components and, in the most severe cases, loss of neuronal polarity. The absence of Prickle2 disrupts the organization of the MT and actin cytoskeleton at the AIS and impairs AP firing in cultured neurons. Our study identifies a critical role for the core PCP protein Prickle2 in establishing neuronal polarity and as a previously unidentified AIS component.

RESULTS

Prickle2 is enriched at the AIS in vivo and in vitro

Using custom-made antibodies (*35*), we identified Prickle2 (Fig. 1, magenta) at the AIS of neurons in several regions of the brain,

[1]Univ. Bordeaux, INSERM, Magendie, U1215, F-33077 Bordeaux, France. [2]Aix-Marseille Université, CNRS, Institut de Biologie du Développement de Marseille, UMR 7288, Case 907, 13288 Marseille Cedex 09, France. [3]Department of Functional Genomics, Vrije Universiteit (VU), Amsterdam, Netherlands. [4]Univ. Bordeaux, CNRS, IMN, UMR 5293, F-33000 Bordeaux, France.
*Corresponding author. Email: mireille.montcouquiol@inserm.fr
†These authors contributed equally to this work.
‡Lead contact.

including, but not restricted to, the cerebral cortex (Fig. 1, A to A″, C to C″, and E) and the hippocampus (Fig. 1, B to B″, D to D″, and F). The labeling was present as early as postnatal day 6 (P6) (Fig. 1, A to B″) and maintained at P14 (Fig. 1, C to D″) and P21 (Fig. 1, E and F). Prickle2 colocalized with AnkG (Fig. 1, green), the AIS master organizer. We validated that the antibody recognizes only the Prickle2 protein and not Prickle1 (fig. S1). Colabeling of 22 days in vitro (DIV22)–dissociated hippocampal neurons with postsynaptic density protein 95 (PSD95) (Fig. 1, G to G″, cyan) confirmed the presence of endogenous Prickle2 in the PSD, consistent with previous reports (Fig. 1G, inset) (*27*, *31*), while much higher levels of the immunofluorescence were detected at the AIS (Fig. 1G, bracket). This enrichment was not described previously and is consistent with a role in regulating AIS formation, maturation, and/or function.

Prickle2 colocalizes with AnkG at the onset of neuronal polarity and AIS formation

We further explored the subcellular distribution of the PCP protein Prickle2 in the earliest stages of neuronal polarity and AIS assembly in rat primary hippocampal neurons. At DIV1 in stage 2 (unpolarized) neurons characterized by many neurites of similar length, we observed the accumulation of endogenous AnkG (Fig. 2, A to A″, green) in many of the neurites (Fig. 2A, asterisks) and systematic overlap with Prickle2 immunolabeling (Fig. 2A, magenta). Prickle2 was also present in the soma of the neuron, unlike AnkG. At the onset of axonal specification in stage 2/3 neurons (DIV1), we detected aggregates of Prickle2 and AnkG in a fragmented profile along the nascent axon (Fig. 2B, arrows). Prickle2 was still present with AnkG after membrane extraction with Triton X-100 detergent at DIV1, suggesting an early and tight association with the cytoskeletal meshwork (Fig. 2, C to C″).

At DIV2, the early stage 3 (polarized) neurons still displayed a fragmented profile of the two proteins. Stimulated Emission Depletion Microscopy (STED) showed that Prickle2 and AnkG colocalized in the proximal axon in a cohesive pattern (Fig. 2D, brackets, inset a), while they colocalized in smaller aggregates in the more distal axon (Fig. 2D, curly bracket, inset b). Even in these distal vesicle-like structures, we detected colocalization of the two proteins (Fig. 2D, inset b). In a more mature stage 3 neuron, the AIS was cohesive, and both Prickle2 and AnkG fully overlapped (Fig. 2, E to E″). Colabeling with TRIM46 (Tripartite Motif Containing 46), an early marker of the proximal axon, revealed only partial colocalization with either AnkG or Prickle2 at the same stage (Fig. 2, F and G).

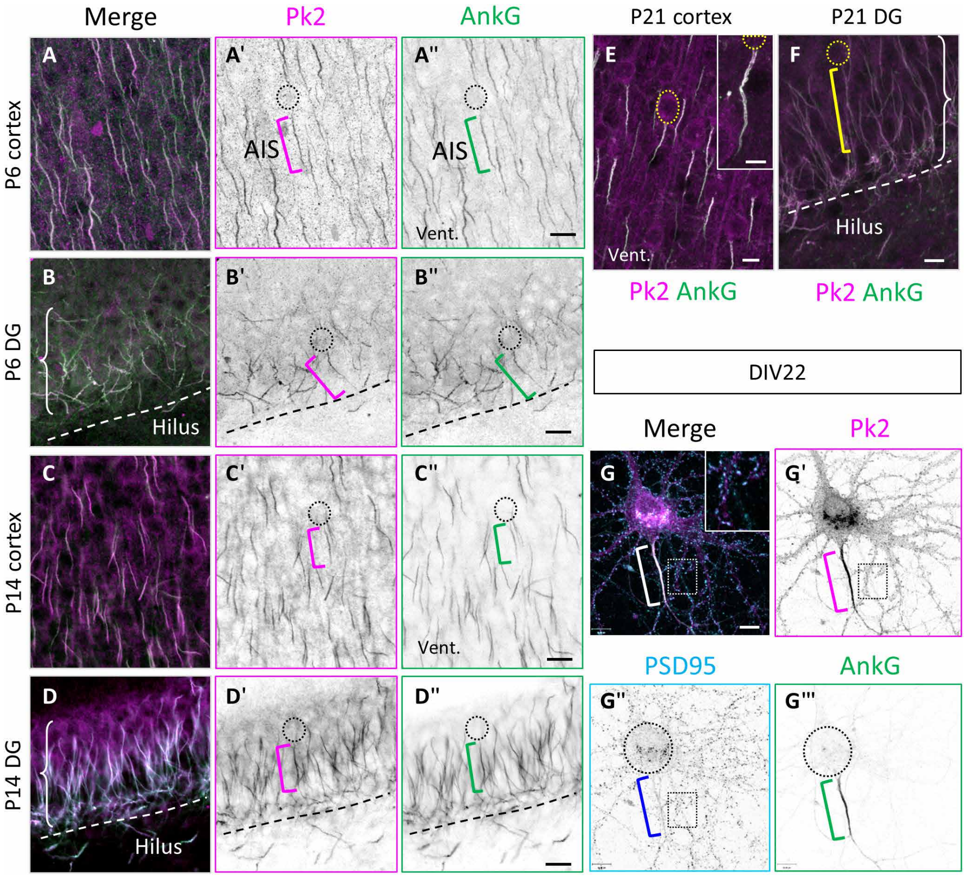

Fig. 1. Pk2 is enriched at the AIS in vivo and in vitro. (**A** to **F**) Illustration of coronal sections of the brain at P6 (A to B″), P14 (C to D″), and P21 (E and F) immunolabeled for Pk2 (magenta) and AnkG (green) in the cortex (A to A″, C to C″, and E) and the dentate gyrus (DG) of the hippocampus (B to B″, D to D″, and F). At every stage, the two proteins fully overlap. Dashed circles outline the cell body, and brackets indicate the AIS. Vent., ventricle. Dotted line delimits the hilus. (**G** to **G‴**) Hippocampal neurons at DIV22 in vitro immunolabeled for Pk2 (magenta), AnkG (green), and the synaptic marker PSD95 (cyan). Pk2 colocalizes with PSD95 [see inset in (G)] and is strongly enriched at the AIS (bracket). All labeling done at least in triplicates. Scale bars, 20 μm (A to D″) and 10 μm (E to G‴).

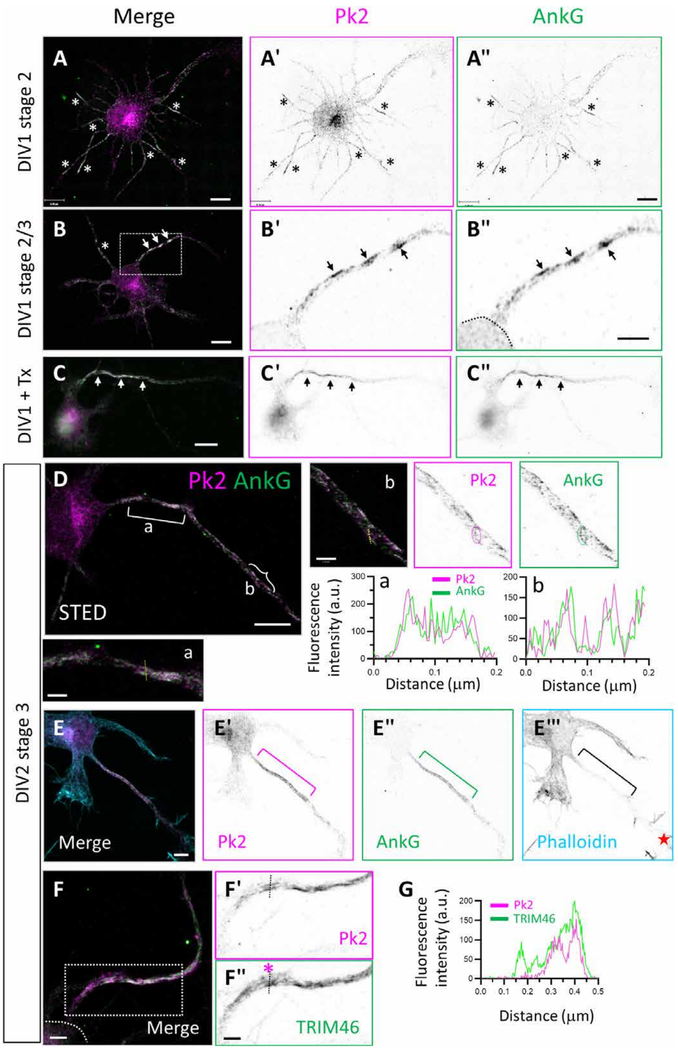

Fig. 2. Pk2 colocalizes with AnkG at the onset of neuronal polarity and AIS formation. (**A** to **A″**) Illustration of a stage 2 neuron at DIV1 with Pk2 (magenta) and AnkG (green) labeling. Pk2 is detected in several immature neurites (asterisks) together with AnkG. (**B** to **B″**) Illustration of a stage 2/3 neuron at DIV1 with Pk2 (magenta) and AnkG (green) labeling. (B to B″) High magnification from (B). Pk2 and AnkG are enriched in clusters in the nascent axon. Arrows point to the protein clusters. Note one neurite in (B) with some Pk2 and AnkG staining (asterisk). (**C** to **C″**) Illustration of a stage 3 neuron at DIV1 with Pk2 (magenta) and AnkG (green) labeling after treating the neurons with detergent extraction [Triton (Tx)] during fixation. Both Pk2 and AnkG labelings are maintained. (**D**) Illustration of a stage 3 neuron at DIV2 with Pk2 (magenta) and AnkG (green) labeling imaged by STED microscopy. Pk2 and AnkG colocalize in clusters or vesicles in the proximal (a) (bracket) and distal (b) (curly bracket) region of the axon. Linescans of (a) and (b) are shown illustrating the parallel profile of the proteins. (**E** to **E‴**) Illustration of a stage 3 neuron at DIV2 with Pk2 (magenta), AnkG (green), and F-actin (phalloidin, cyan) labeling. Pk2 and AnkG define a cohesive and overlapping profile along the forming axon. Star in (E‴) marks the growth cone. (**F** and **G**) Illustration of a stage 3 neuron at DIV2 with Pk2 (magenta) and TRIM46 (green) in the nascent axons. The linescans (G) as illustrated in (F) and (F′) (asterisk) show only a partial overlap of Pk2 and TRIM46. All labeling done at least in triplicates. Scale bars, 8 μm (A and B), 4 μm (B′, B″, and F to F″), 10 μm (C to C′), 8 μm (D), 2 μm (D, insets), and 5 μm (E to E‴).

From DIV4 and on, immunolabeling showed that Prickle2 and AnkG fully colocalized in a cohesive pattern as the AIS matured and shortened (Fig. 3, A to C). Linescan analysis confirmed an overlap at all stages (Fig. 3, D to F). Together, our data suggest that Prickle2 is one of the earliest partners of AnkG in the developing axon.

Prickle2 binds to AnkG, leading to MT bundling

The colocalization of Prickle2 with AnkG at the earliest stages of neuronal polarization suggests that the proteins might interact. We tested this interaction using coimmunoprecipitation (coIP) and glutathione *S*-transferase (GST) pulldown assays. Because of the large size of AnkG480 (called giant AnkG), we restricted our coIP experiments to the 270- and 190-kDa isoforms (fig. S2A). We coimmunoprecipitated Flag-hPrickle2 with both AnkG270–GFP (green fluorescent protein) and AnkG190-GFP (Fig. 4, A to C). We were also able to coIP AnkG270-GFP with Flag-hPrickle2 (fig. S2B). Our results are consistent with Prickle2 interacting with the N-terminal region of AnkG, similar to many diverse proteins also interacting with AnkG (*36*). We confirmed this interaction by coIP between an N-terminal version of AnkG containing only the Ankyrin repeats (see Materials and Methods) and Flag-hPrickle2 (Fig. 4D). We next generated GST constructs from different regions of Prickle2. Prickle2 contains three domains that are conserved across species, and these include the N-terminal PET (Prickle, Espinas, and Testin) and LIM (Lin-11, Isl-1, and Mec-3) domains, as well as the C2 domain, near the C terminus of the protein (Fig. 4E) (*37*, *38*). Using these constructs, we performed GST pulldown assays. We detected two domains of Prickle2 required for the interaction: a region corresponding to the LIM domain (amino acids 127 to 323) and a region corresponding to the C-terminal region of the protein that contains the C2 domain (amino acids 585 to 844), which appeared to be the strongest binding site (Fig. 4F). Last, we confirmed this interaction in vivo by

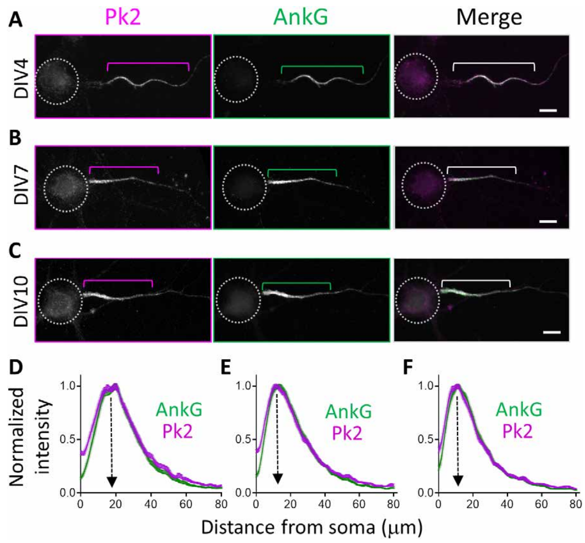

Fig. 3. Pk2 and AnkG colocalize during and after AIS formation. (**A** to **C**) Illustration of Pk2 (magenta) and AnkG (green) labeling at DIV4 (A), DIV7 (B), and DIV10 (C), respectively. Dotted circles outline the cell body, and brackets indicate the AIS. (**D** to **F**) Linescans of normalized fluorescence intensity at the AIS of Pk2 and AnkG at DIV4 (D), DIV7 (E), and DIV10 (F). The two proteins colocalize at the AIS at all analyzed stages. Values are normalized to the maximum intensity of each marker. Scale bars, 10 μm. Statistics, Table 1.

Table 1. Overview statistics.

Figure	Data type	Sample size (N and n) and P value	Statistical test
1	Immunolabeling	$N \geq 3$ independent experiments	
2	Immunolabeling	$N \geq 3$ independent experiments	
3	Means ± SEM (D to F)	$N \geq 3$ independent experiments	
		$n = 77$ neurons for DIV4, $n = 87$ for DIV7, and $n = 66$ for DIV10	
4	Immunoblot, 4 (B to G)	$N = 3$ independent experiments for (B), (C), (F), and (G); $N = 2$ for (D)	
	Immunolabeling, 4 (H to L‴)	$N = 3$ independent experiments	
	Median and upper and lower quartiles, 4K	$N = 3$ independent experiments	Unpaired t test with Welch's correction
		Total cells quantified: $n = 195$ (Pk2 1 μg + AnkG480), $n = 292$ (Pk2 2 μg + AnkG480), $n = 218$ (Pk2 3 μg + AnkG480), $n = 120$ (Pk2 1 μg + AnkG190), $n = 120$ (Pk2 2 μg + AnkG190), and $n = 120$ (Pk2 3 μg + AnkG190)	
		Between Pk2–1 μg and Pk2–2 μg, $*P < 0.05$	
		Between Pk2–2 μg and Pk2–3 μg, $P = 0.6134$	
5	Immunolabeling, 5 (A to D‴)	$N \geq 3$ independent experiments	
	Means ± SEM, 5E	$N \geq 3$ independent experiments	
		$n = 220$ neurons for control and $n = 344$ for shPk2A	
		$N = 2$ independent experiments, $n = 88$ neurons for control, and $n = 72$ for shPk2B	
	Median and upper and lower quartiles, 5 (F to K)	$N = 3$ independent experiments	Mann-Whitney's test
		(F and G) Pk2 and AnkG: $n = 136$ neurons for control and $n = 245$ for shPk2A.	
		(H) β4-spectrin: $n = 73$ neurons for the control and $n = 75$ neurons for shPk2	
		(I) NF-186: $n = 60$ neurons for control and $n = 82$ for shPk2	
		(J and K) Pk2 and AnkG: $N = 136$ neurons for control and $n = 245$ for shPk2A.	
		Between control and shPk2, $****P < 0.0001$ for (F) to (I)	
		Between control and shPk2 1 AIS and between control and shPk2 Multi AIS (J and K): $****P < 0.0001$ for (F) to (I)	
	Immunolabeling, 5 (L to N″)	$N \geq 3$ independent experiments	

continued on next page

Figure	Data type	Sample size (*N* and *n*) and *P* value	Statistical test
6	Immunolabeling, 6 (A and B)	$N \geq 3$ independent experiments	
	Median and upper and lower quartiles, 6C	$N = 3$ independent experiments	Welch's *t* test
		Proximal AIS: $n = 35$ neurons for control and $n = 39$ for shPk2	
		Distal AIS: $n = 35$ neurons for control and $n = 38$ for shPk2	
		Between control and shPk2 for proximal and distal AIS: ****$P < 0.0001$	
	Immunolabeling, 6 (D and E)	$N = 2$ independent experiments	
		$n = 5$ neurons for control and 6 for shPk2 were imaged	
	Immunolabeling, 6 (F and G)	$N \geq 3$ independent experiments	
	Median and upper and lower quartiles, 6H	$N = 3$ independent experiments,	Mann-Whitney's test
		$n = 46$ neurons quantified for control and $n = 37$ for shPk2	
		Between control and shPk2, ****$P < 0.0001$	
	Immunolabeling, 6 (I to J″)	$N = 3$ independent experiments	
	Means ± SEM, 6K	$N = 3$ independent experiments, $n = 35$ neurons for control, and $n = 29$ neurons for shPk2	Mann-Whitney's test
		Between control and shPk2, ****$P < 0.0001$	
	Means ± SEM, 6N	$N = 4$ independent experiments	Two-way ANOVA test
		$n = 28$ neurons (axons and dendrites) for control and $n = 21$ neurons (axons and dendrites) for shPk2	
		Between control and shPk2, ****$P < 0.0001$	
	Median and upper and lower quartiles, 6O	$N = 4$ independent experiments	One-way ANOVA test
		$n = 28$ neurons (axons and dendrites) for control and $n = 21$ neurons (axons and dendrites)	
		For axon control versus axon shPk2, **$P < 0.01$	
		For dendrite control versus dendrite shPk2, ****$P < 0.0001$	
	Median and upper and lower quartiles, 6P	$N = 4$ independent experiments	One-way ANOVA
		$n = 28$ neurons (axons and dendrites) for control and $n = 21$ neurons (axons and dendrites)	
		For axon control versus neurite shPk2, *$P < 0.05$	
7	Immunolabeling, 5 (B and C)	$N = 3$ independent experiments	
	Immunolabeling, 5 (D to E‴)	$N = 3$ independent experiments	
	Means ± SEM, 7F	$N = 6$ brains from two independent experiments	Mann-Whitney's test
		$n = 36$ analyzed neurons for shPk2 and $n = 32$ surrounding neurons	
		For nontransfected versus shPk2, **$P < 0.01$	
	Means ± SEM, 7G	$N = 3$ brains from two independent experiments	*t* test with Welch's correction
		$n = 35$ neurons for shPk2 and $n = 77$ surrounding neurons	
		For nontransfected versus shPk2, ***$P < 0.001$	
	Median and upper and lower quartiles, 7H	$N = 3$ brains from 2 independent experiments	*t* test with Welch's correction
		$n = 19$ shPk2 neurons and $n = 77$ surrounding neurons	
		For nontransfected versus shPk2, ****$P < 0.0001$	

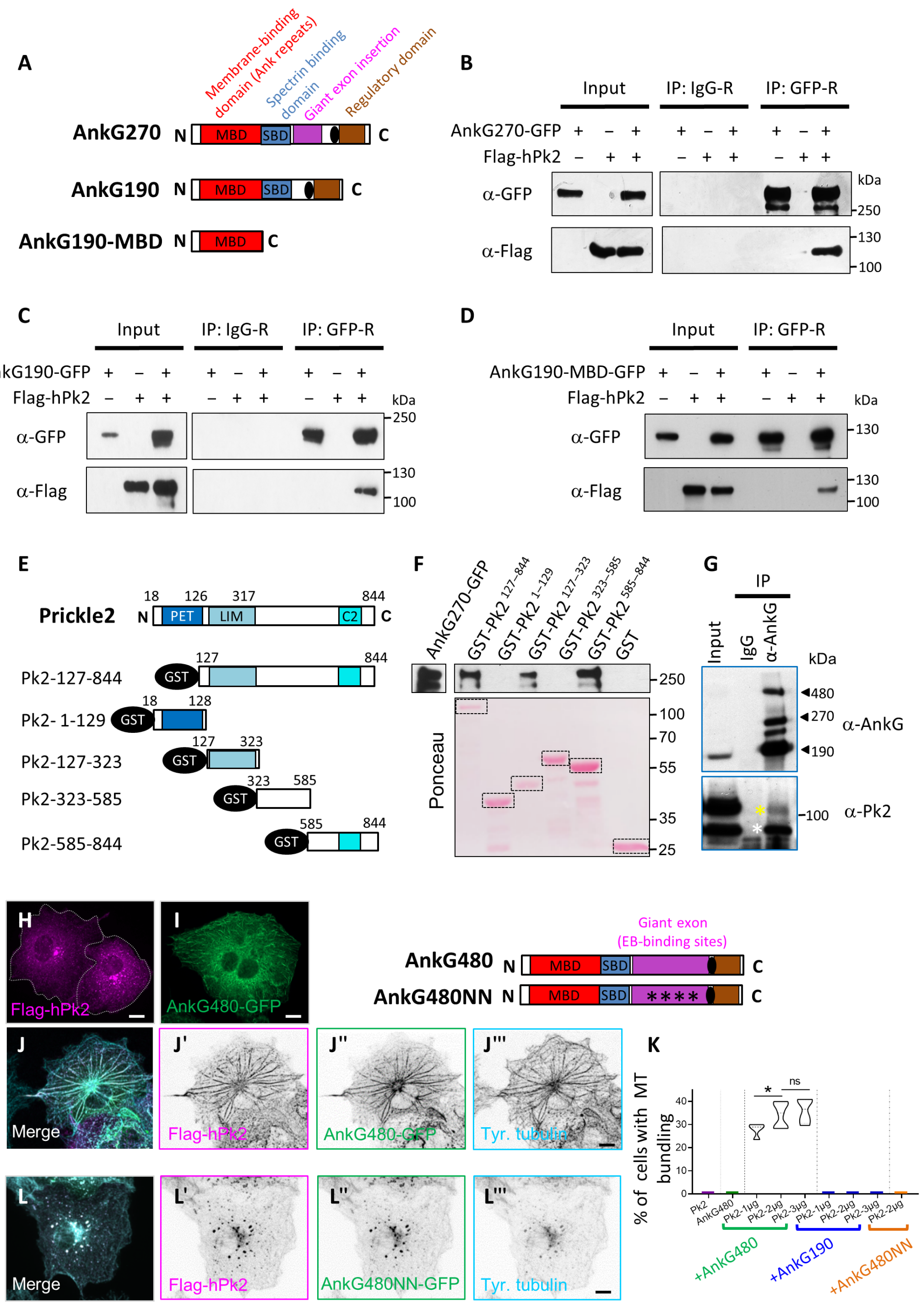

Fig. 4. Pk2 binds to AnkG, leading to MT bundling. (**A**) Schematic of the AnkG270, the AnkG190 isoform domains of AnkG, and the short AnkG-MBD. N, N terminus; C, C terminus; MBD, membrane-binding domain; SBD, spectrin-binding domain. (**B** and **C**) coIP of AnkG with Flag-Pk2. Both AnkG isoforms interact with Pk2 but not with immunoglobulin G (IgG)–R serum. (**D**) coIP of the short AnkG-MBD with Flag-Pk2. (**E**) Schematic of the full-length Pk2 protein with the different domains and the corresponding amino acid numbers and the GST constructs used. (**F**) GST pulldown experiments showing that Pk2 interacts with AnkG via the LIM and the C-terminal domains, the latter including the C2 region. (**G**) Pk2 coimmunoprecipitates with AnkG in vivo but not with IgG control serum (Ig). Asterisks indicate the two bands immunoprecipitated. (**H** and **I**) COS7 cells transfected with Flag-Pk2 (H) or AnkG480-GFP (I). (**J** to **J‴**) Flag-Pk2 (magenta) and AnkG480-GFP (green) promote the bundling of MTs, strongly immunoreactive to tyrosinated tubulin [cyan in (J‴)]. Note the relocalization of Pk2 and AnkG to the MT bundles. (**K** to **L‴**) MT bundling is lost upon coexpression of Flag-Pk2 with AnkG480NN, an AnkG480 construct that can no longer bind to MTs. Scale bars, 10 μm. Statistics, Table 1.

performing coIP of Prickle2 with AnkG in P21 rat cortical lysates, with an expected 100-kDa band observed for full-length Prickle2 and an additional uncharacterized 70-kDa band (Fig. 4G). Together, our results establish Prickle2 as a novel interacting partner of AnkG.

Our data support the hypothesis that Prickle2 is an early interacting partner of AnkG in the nascent axon at the time of AIS assembly. Since MTs are critical in the early phases of axonal specification and AIS establishment, we used a cell-based assay (*17*) to evaluate the interplay of Prickle2 with MTs in the presence of

GFP-tagged AnkG. Single transfections of Flag-hPrickle2 or the giant AnkG isoform (AnkG480-GFP) in COS-7 cells resulted in the formation of cytosolic vesicles and/or aggregates for Prickle2 (Fig. 4H) and a cytosolic and sometimes comet-like pattern for AnkG480 (Fig. 4I). Coexpression of each AnkG isoform with Flag-Prickle2 resulted in colocalization of the proteins in cytosolic clusters (fig. S2, C to E). In a proportion of cells, we observed a marked relocalization of Prickle2 and AnkG480 along thick stretches that were immunoreactive for tyrosinated tubulin and resembling thick bundles of MTs (Fig. 4, J to J‴). AnkG480-GFP and Flag-Prickle2 were distributed along these MT bundles, and notably, AnkG480-GFP was no longer observed in a comet-like pattern at the tips of MTs. These MT bundles often extended from the center of the cell (aster-like pattern) but were also sometimes circular at the cell periphery or fragmented (fig. S2, F to F‴). Our data are consistent with AnkG acting as an early MT bundler as suggested by previous studies on AnkG mutants (*15*, *39*, *40*), upon binding to Prickle2. Transfection of graded levels of Flag-hPrickle2 cDNA with a constant amount of AnkG480-GFP resulted in increasing numbers of cells exhibiting an MT bundle profile, reaching a plateau at 3 μg of cDNA per well (27.8 ± 1.48% for 1 μg per well, 34.2 ± 2% for 2 μg per well, and 36 ± 2.7% for 3 μg per well) (Fig. 4K). This phenotype was never observed in cells transfected with Flag-Prickle2 or AnkG480-GFP alone or with AnkG190-GFP (Fig. 4K). Prickle1 also promoted AnkG-dependent MT bundling, although with less efficiency than Prickle2 (14.9 ± 3%) (fig. S2, G to G‴). Since the bundling was restricted to the giant AnkG480 that differs noticeably from the two other AnkG isoforms by the presence of MT-EB sites, we used a previously characterized AnkG480 variant with mutated EB1- and EB3-binding sites, AnkG480NN (*16*). AnkG480NN behaved similarly to AnkG190, indicating the requirement for MT-EB sites for bundling (Fig. 4, L to L‴ and K). Together, our results show that Prickle2 is an interacting partner of AnkG, mediating its recruitment along the MT lattice and promoting AnkG-dependent MT bundling activity.

Prickle2 depletion in vitro and in vivo disrupts neuronal polarity and AIS formation and reduces axonal excitability

Next, we examined the effect of Prickle2 depletion on the AIS and axons in cultured neurons at DIV7. We used a previously validated short hairpin RNA (shRNA) against Prickle2 (shPrickle2A; Fig. 5, A to E) (*41*) and a new shRNA (shPrickle2B; Fig. 5E). Both shPrickle2 constructs effectively depleted Prickle2 at the AIS, as assessed by immunolabeling, in comparison to control-shRNA–transfected neurons (Fig. 5, A to D‴). Unless otherwise specified, we used shPrickle2A for the subsequent experiments. The depletion of Prickle2 in neurons at the time of plating resulted in a net decrease in relative Prickle2 (Fig. 5F) and AnkG immunofluorescence levels (Fig. 5G) and three structural phenotypes that correlated with the degree of Prickle2 down-regulation (Fig. 5, B to E). In the least severe phenotype, 22.2 ± 9.4% of Prickle2-depleted neurons (31.2 ± 2.6% for shPrickle2B) maintained their polarity with a single AnkG-positive axon (single-AIS/axon phenotype) that was sometimes fragmented (Fig. 5, B to B‴). In these neurons, Prickle2 levels were reduced by 62% (Fig. 5J), and AnkG levels were reduced by 57% [intensity levels were $4.14 \pm 0.2 \times 10^5$ arbitrary units (a.u.) for Prickle2 and $6.59 \pm 0.3 \times 10^5$ a.u. for AnkG compared to the control values of $10.8 \pm 0.3 \times 10^5$ a.u. for Prickle2 and $15.5 \pm 0.5 \times 10^5$ a.u. for AnkG] (Fig. 5K). For shPrickle2B, we observed a 60% decrease in Prickle2 levels and a 37% decrease in AnkG levels. In the second and most common phenotype we called multiple-AIS/axon phenotype, we observed an alteration of neuronal polarity. Approximately two-thirds of the Prickle2-depleted neurons had more than three neurites immunoreactive for AnkG/Prickle2 (multiple-AIS/axon phenotype) (63.9 ± 14.4% for shPrickle2A and 49.3 ± 3.1% for shPrickle2B). This multiple-AIS/axon phenotype correlated with a 75% decrease in Prickle2 levels (Fig. 5J) and a 71% reduction in AnkG levels compared to the control (Prickle2 levels of $2.72 \pm 0.1 \times 10^5$ a.u. and AnkG levels of $4.48 \pm 0.2 \times 10^5$ a.u.) (Fig. 5K). For shPrickle2B, we observed a 70% decrease in Prickle2 levels and a 54% decrease in AnkG levels. In the most severe phenotype, there was a complete loss of neuronal polarity with 13.9 ± 6.5% of Prickle2-depleted neurons with no AnkG/Prickle2 labeling (19.5 ± 0.5% for shPrickle2B, no-AIS/axon phenotype) (Fig. 5E).

Consistent with the role of AnkG as master organizer of the AIS (*1*–*3*), relative labeling for other structural AIS components was also reduced in Prickle2-depleted neurons at DIV7, including a 72% decrease in β4 spectrin ($32.1 \pm 1.13 \times 10^5$ a.u. intensity for the controls versus $9.08 \pm 0.3 \times 10^5$ a.u. for shPrickle2-transfected cells; Fig. 5H and fig. S3, A to B″) and 56% decrease in NF-186 ($18.6 \pm 0.75 \times 10^5$ a.u. intensity for the controls versus $8.28 \pm 0.41 \times 10^5$ a.u.; Fig. 5I and fig. S3, C to D″). Showing the codependence of AnkG and Prickle2 in AIS formation, only 7.4 ± 3.2% of neurons targeted with shRNA for AnkG were weakly positive for AnkG and Prickle2, compared to 97.8 ± 1.8% of control neurons (fig. S3, E to G). We could detect the fragmented AIS (Fig. 5, L to M″) and no AIS (Fig. 5, N to N″) phenotypes as early as DIV3, demonstrating the early impact of Prickle2 depletion on neuronal polarity. In these neurons, MAP2 (microtubule associated protein 2) expression was overall reduced while still excluded from the distal axon when AnkG labeling was present (arrowheads in M to M″). On the basis of these results, we conclude that Prickle2 is necessary for neuronal polarity and for AnkG recruitment and/or stabilization at the AIS and that AnkG is reciprocally required for Prickle2 recruitment to the AIS in vitro.

Early Prickle2 depletion disrupts cytoskeletal organization at the AIS

We focused on analyzing the most common phenotype (multiple AIS/axons) present in Prickle2-depleted neurons. We observed a decrease in thickness of the proximal AIS region (2.3 ± 0.12 μm for control and 1.4 ± 0.07 μm for shPrickle2) and in a more distal AIS region (0.81 ± 0.04 μm for control and 0.52 ± 0.02 μm for shPrickle2) compared to the same regions in controls (Fig. 6, A to C). This result is consistent with a disruption of the maturation of the AIS that depends on the cytoskeleton organization (*9*, *42*, *43*). To confirm this, we first looked at F-actin organization. A periodic distribution of F-actin is observed in most neuronal processes, but it is best organized in axons (*44*). In Prickle2-depleted neurons, F-actin periodicity at the AIS was strongly disrupted with an overall increased periodicity (from 189 ± 7 nm in controls to 365 ± 46 nm in depleted neurons) and decreased number of actin rings, although it was present in control neurons (Fig. 6, D and E, and fig. S4, A to C). Next, we evaluated the MAP and MT cross-linker TRIM46. Results show that Prickle2-depleted neurons exhibited a 40% decrease in relative immunofluorescence levels of TRIM46 ($13.09 \pm 1.01 \times 10^5$ a.u. for the control and $7.80 \pm 0.82 \times 10^5$ a.u. for shPrickle2), similar to the decreased TRIM46 fluorescent intensity reported after

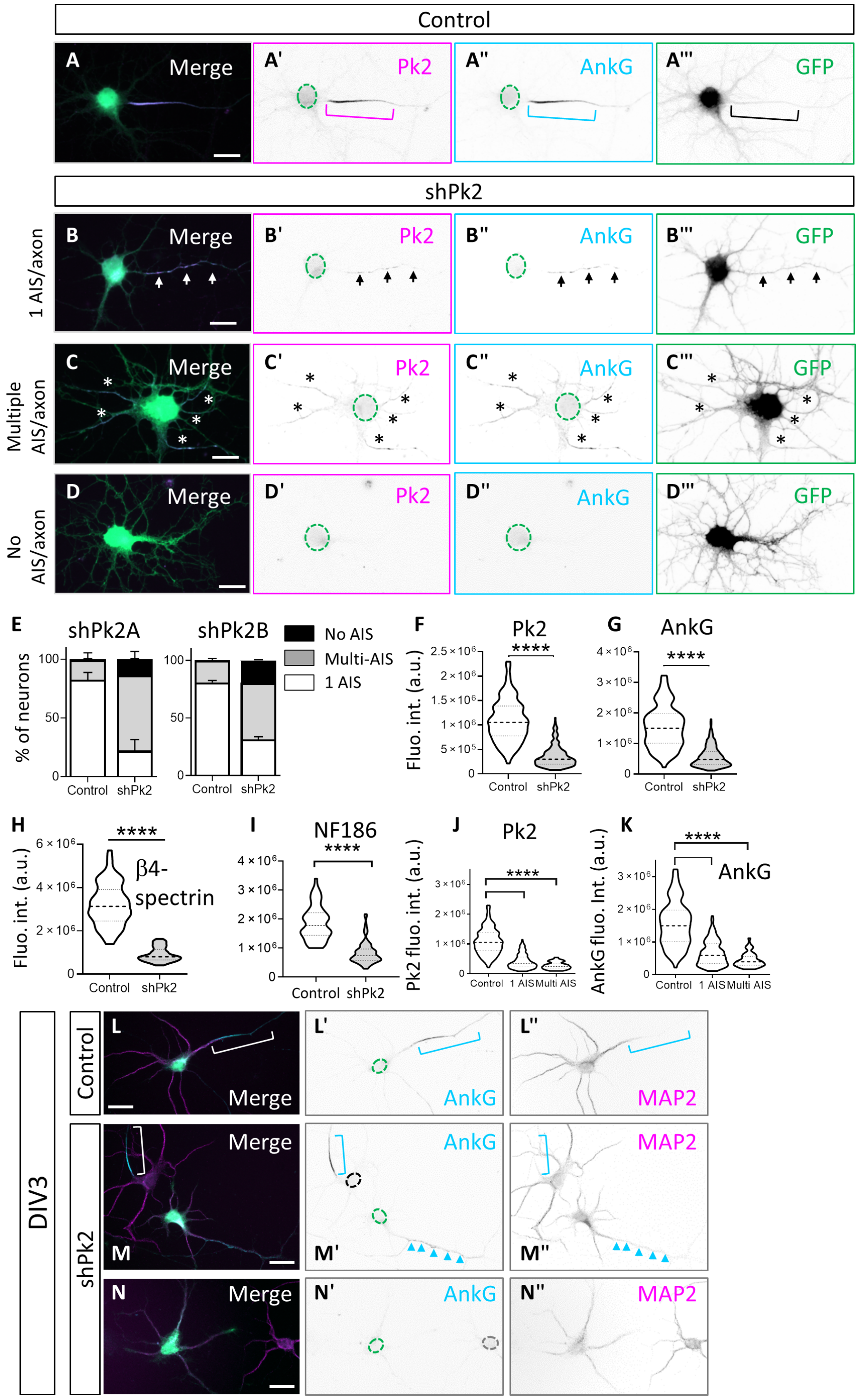

Fig. 5. Pk2 regulates axonal establishment and AIS formation. (**A** to **A‴**) Illustration of a DIV7 neuron transfected with a control plasmid and labeled for Pk2 (magenta) and AnkG (cyan). Dashed circles outline the cell body, and bracket indicates the AIS. (**B** to **B‴**) Illustration of a DIV7 Pk2-depleted neuron with a single-AIS/axon phenotype. Note the decrease in AnkG labeling. Arrows point at the fragmented AIS. (**C** to **C‴**) Illustration of a DIV7 Pk2-depleted neuron with a multiple-AIS/axon phenotype. The various AnkG-positive processes are indicated by the asterisks. (**D** to **D‴**) Illustration of a DIV7 Pk2-depleted neuron with a no-AIS/axon phenotype. (**E**) Percentages of neurons per phenotype with shPk2A and shPk2B. (**F** to **I**) Quantifications of fluorescence intensity levels of Pk2 in shPk2A neurons (from one-AIS and multiple-AIS phenotypes) show strong down-regulations of Pk2 (F) and AnkG (G) levels. (**J** and **K**) Phenotype severity in neurons as a function of Pk2 and AnkG levels. (**L** to **M″**) At DIV3, MAP2 immunolabeling is decreased in the neurites of Pk2-depleted neurons but excluded from the distal region of the neurite with fragmented AnkG labeling (arrowheads) (M to M″) compared to control (L to L″) or a nontransfected neuron nearby (M to M″) (bracket). In neurons with no AnkG staining, MAP2 is present in all neurites (**N** to **N″**). Dashed circles outline the cell body and brackets the AIS. Scale bars, 10 μm (A to D) and 20 μm (L to N). Statistics, Table 1.

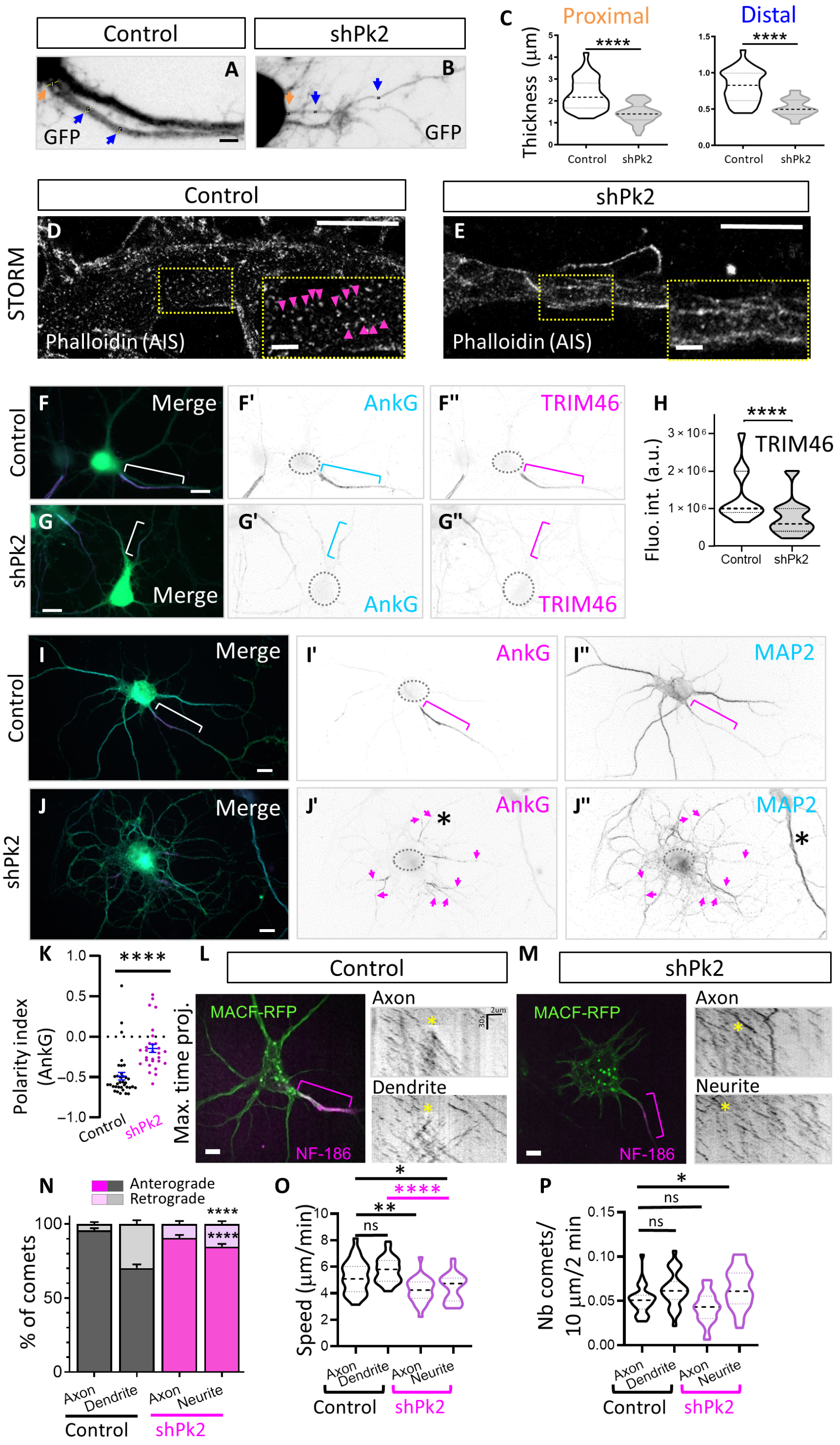

Fig. 6. Pk2 depletion impairs the AIS cytoskeleton network. (**A** and **B**) Immunolabeling for GFP in control (A) and Pk2-depleted neurons (B). (**C**) Average thickness of AnkG-positive axons. In both proximal [orange arrows in (A) and (B)] and distal [blue arrows in (A) and (B)], Pk2 depletion leads to a severe reduction in thickness. (**D** and **E**) STORM imaging of F-actin shows a loss of actin periodicity at the AIS of Pk2-depleted (E) neurons compared to control (D) at DIV7. (**F** to **G″**) TRIM46 immunolabeling is reduced at the AIS of Pk2-depleted neurons (G to G″) compared to control (F to F″). (**H**) Fluorescence intensity levels of TRIM46 at the AIS from neurons in (F) to (G″). (**I** to **J″**) MAP2 immunolabeling is decreased in Pk2-depleted neurons (J to J″) compared to control (I to I″) and restricted to the somatodendritic domain at DIV7. Note the MAP2 staining of the dendrite of a nontransfected cell comparison in (J″) (asterisk). Arrows indicate the multiple AISs. (**K**) PI of AnkG in DIV7 neurons. (**L** and **M**) Illustration of live neurons expressing MACF-RFP (green) and labeled with fluorescent-tagged NF-186 antibody (magenta). Right: Kymographs of control axons and dendrites and shPk2 NF-186–positive (axons) and NF-186–negative neurites at DIV7. Yellow asterisks indicate the severing position. (**N**) Percentage and identity of comets in axons and dendrites after laser cut. (**O**) MT growth speed in control (black) versus shPk2 (magenta) neurons, in axon and dendrites. (**P**) Number of comets displayed by control (black) and shPk2 (magenta) neurons, in axon and dendrites. Scale bars, 4 µm (A and B), 2 µm and 0.5 µm [for higher magnification (D and E)], and 10 µm (F to G″, I to J″, L, and M). Bracket indicates the AIS. Statistics, Table 1.

AnkG depletion (Fig. 6, F to H) (*45*). We also observed a severe reduction in neurofilament content in Prickle2-depleted neurons compared to controls (fig. S4, D to E″). At DIV7 (Fig. 6, I to J″), MAP2 levels were reduced, similar to that at DIV3, and the protein maintained a dendritic distribution and did not invade the AnkG-positive axons (Fig. 5N). Together, these results are consistent with a disruption of the maturation of the AIS that depends on the cytoskeleton organization.

We determined a polarity index (PI) (*45*) based on the presence of AnkG in one or multiple neurites (see Materials and Methods). The PI shifted from ~−0.5 ± 0.05 in controls, a value indicative of AnkG restricted to one axon, to a PI of ~−0.14 ± 0.05 in Pk2-depleted neurons, indicative of AnkG less polarized and distributed across multiple axons (Fig. 6K). Our results suggest that the multiple-axon phenotype results from a loss of restriction of AnkG in one unique neurite at the time of axonal specification.

To further evaluate this alteration of neuronal polarity, we explored MT polarity and dynamics. When neuronal polarity is established, MTs are uniformly arrayed in axons and have mixed orientation in dendrites. This organization has strong functional implications as it is the basis for directed transport throughout the neuron (*46*–*49*). We used this polar organization of MT to assess whether their orientation and dynamics were different in control from those in Prickle2-depleted neurons via a laser-severing approach (*50*). Live labeling using a fluorescently tagged Neurofascin-186 antibody (NF-186) revealed a clear accumulation of the protein at the AIS in control neurons (Fig. 6L, bracket) and a much weaker labeling in Prickle2-depleted neurons, consistent with the 40% AnkG reduction in the one-axon phenotype (Fig. 6M, bracket, as reported in Fig. 5I). We limited our analysis to axons clearly identifiable as NF-186 positive, and we defined all of the remaining extensions as "neurites," allowing us a comparison between axons and dendrites. We used MACF43 (Microtubule Actin Crosslinking Factor 43)–red fluorescent protein (RFP), a 43–amino acid fragment of the C-terminal portion of the MT-actin cross-linking factor MACF that binds to the plus end of MTs (*51*), to image the growing plus end of MTs. To determine the orientation of both dynamic and stable MT populations, we used a laser-severing approach (severing position indicated by a yellow asterisk) and tracked the direction of MT plus-end regrowth. By analyzing corresponding kymographs, we observed that control neurons displayed a typical MT orientation, with axons containing mainly plus-end out MTs (95.3% of anterograde regrowth events), and dendrites having a more mixed orientation (70.2% of plus-end out and 29.8% of minus-end out) (Fig. 6N). Axons of Prickle2-depleted neurons showed a slightly reduced plus-end out MT compared to controls (90.9% of anterograde regrowth). Notably, the MT orientation in other neurites that we could not identify as axon based on NF-186 labeling resembled that of axons in controls and was statistically different from dendrites in controls [84.6% plus-end out MT; $P < 0.0001$, two-way analysis of variance (ANOVA)] (Fig. 6N). In addition to the alteration of MT orientation, we observed an overall reduced speed of MT growth both in the axons and neurites in Prickle2 knockdown neurons (Fig. 6O). In contrast, the number of comets in the axon or in the neurites was not statistically different from that in Prickle2-depleted neurons, although slightly reduced in axons (1.28 to 1.06 comets/μm per 2 min in control versus knockdown neurons, $P = 0.29$) (Fig. 6P). These data confirm that Prickle2 depletion leads to a loss of dendritic identity in favor of axonal features.

Together, these results support the hypothesis that in Prickle2-depleted neurons, the multiple-axon phenotype results from a loss of restriction of AnkG in one unique neurite at the time of axonal specification. The distribution of AnkG in multiple neurites correlates with the specification of multiple axons, by affecting the organization of the cytoskeleton.

In vivo deletion of Prickle2 impairs AIS formation in neocortical neurons

We confirmed the consequences of Prickle2 depletion on AnkG in vivo by performing in utero electroporation (IUE) of shPrickle2 or the control in embryonic day 16.5 (E16.5) rat embryos (Fig. 7A) and analyzing their cerebral cortex at P5 and P6. Prickle2-transfected neurons were identified in the neocortex with GFP labeling (Fig. 7, B and C). In all of our samples, Prickle2 depletion resulted in a proportion of GFP-positive neurons that were unable to migrate and remained close to the ventricular region (Fig. 7, B and C, green dashed oval). These neurons might represent the most severe phenotype of Prickle2 depletion, with an early and complete loss of polarity, preventing migration.

We evaluated the levels of Prickle2 and AnkG in the same sample under the same conditions by first validating that the control construct did not affect the levels of either Prickle2 or AnkG (fig. S5, A and B). We observed no difference in Prickle2 AIS labeling intensity between the surrounding (mean intensity, 276.6 ± 11.68 a.u.) and GFP-positive (mean intensity, 293.7 ± 23.92 a.u.) neurons. Similar results were found for AnkG, with values of 252.3 ± 11.98 a.u. for the surrounding neurons and 267.7 ± 25.03 a.u. for GFP-positive neurons, confirming that the construct is a suitable control.

Only 3 ± 1.92% of the shPrickle2-GFP–positive neurons had some remaining Prickle2 immunolabeling compared with the nonelectroporated surrounding neurons, confirming the knockdown efficiency of the construct (Fig. 7, E to E‴ and F). Only 55.7 ± 0.75% of shPrickle2-GFP–positive neurons presented some AnkG labeling, which was reduced by 42% compared to nonelectroporated neurons (the AnkG intensity for the surrounding neurons was 368.2 ± 19.12 versus 213.7 ± 20.22 a.u. for GFP-positive neurons) (Fig. 7, E to E‴, G, and H). We were unable to evaluate the length of the AIS because of the overall severity of the AnkG decrease, although they appeared often shorter. On the basis of these results, we conclude that Prickle2 is necessary for neuronal polarity and for AnkG recruitment and/or stabilization at the AIS of cortical neurons in vivo.

Prickle2 depletion alters neuronal excitability

The AIS as the site of AP initiation is critically dependent on AnkG-mediated voltage-gated sodium channel (Na_v) channel enrichment. Previous work has established that AnkG knockout neurons lack an AIS and have reduced sodium channel clustering, leading to a reduced firing rate (*52*). We thus investigated whether the observed structural changes of the AIS could affect neuronal excitability. Using whole-cell patch-clamp recordings, we evaluated the effects of Prickle2 depletion on neuronal excitability in dissociated neurons at DIV8 and assessed the intrinsic properties of control and Prickle2-depleted neurons. Although the resting membrane potential was unchanged in Prickle2-depleted neurons (see Table 2), input resistance was increased, indicating less open ion channels at resting membrane potential or suggesting neuronal maturation delay. We next evaluated the AP firing properties of these neurons using 800-ms-duration step-current injections (Fig. 8A). All neurons

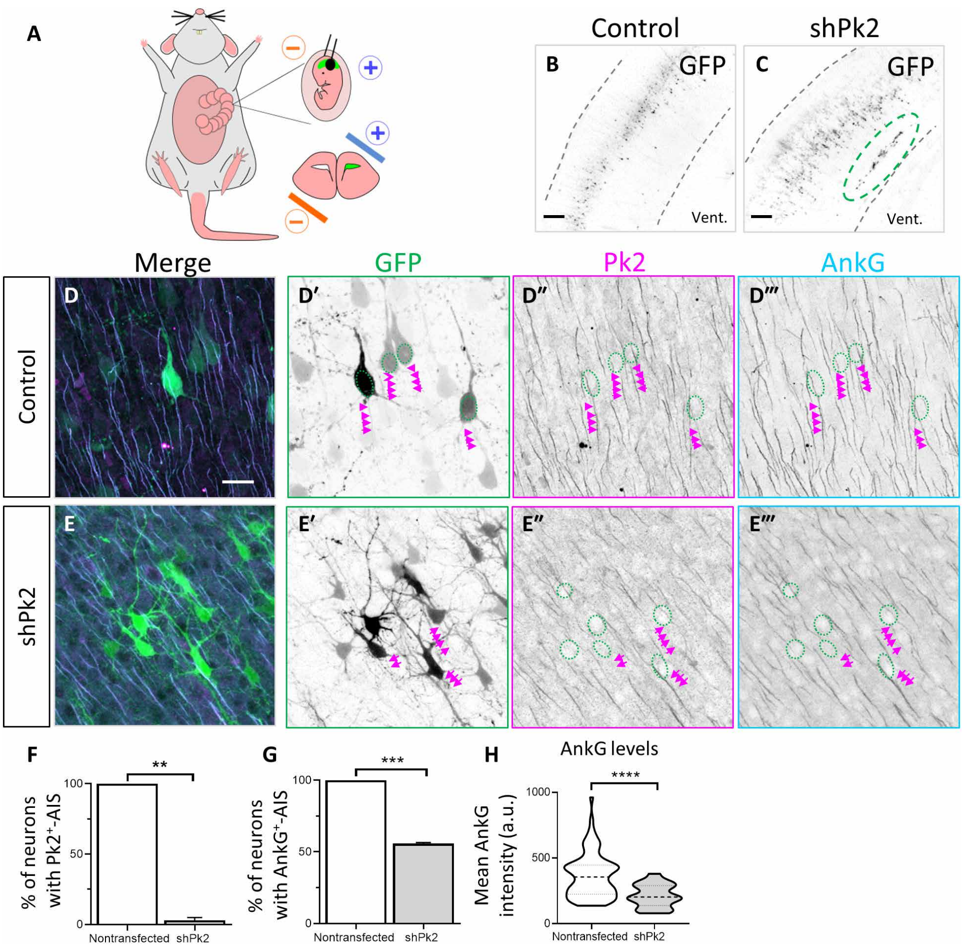

Fig. 7. In vivo deletion of Pk2 impairs AIS formation in neocortical neurons. (**A**) Schematic of IUE at E16.5 in rat embryos. (**B** and **C**) Low magnifications of coronal sections from P6 control and shPk2-electroporated brains immunolabeled for anti-GFP to identify GFP-positive neurons. In (C), note that some Pk2-depleted neurons remain stuck close to the ventricular zone (vent) (circled in green), while in the control, they all migrate dorsally. (**D** to **E‴**) In GFP-positive control neurons (D to D‴) (green circles), Pk2 and AnkG colocalize at the AIS (arrows), while in Pk2-depleted neurons, almost no Pk2 or AnkG remains (E to E‴). (**F**) Percentage of Pk2-positive neurons in control and shPk2 brain sections as compared to the surrounding, nonelectroporated cells. (**G**) Percentage of AnkG-positive neurons in control and shPk2 brain sections as compared to the surrounding, nonelectroporated cells. (**H**) AnkG levels at the AIS in the shPk2 neurons with a positive AnkG labeling. Scale bars, 200 μm (A to C) and 20 μm (D to E‴). Statistics, Table 1.

from the control group generated APs; in contrast, only 10 of 12 neurons from the shPrickle2-expressing group fired APs (Fig. 8B). An analysis of the current-frequency curve (Fig. 8C) showed an unchanged current threshold and slope but a reduction in the maximum firing frequency (Fig. 8D). Analysis of single APs showed that Prickle2-depleted neurons had a depolarized AP threshold (Fig. 8E). These functional data were supported by decreased sodium channel clustering at the AIS in Prickle2-depleted neurons at DIV8 (Fig. 8, F to H). Relative labeling for Na_v channels was decreased by 60% in Prickle2-depleted neurons ($16.67 \pm 0.76 \times 10^5$ a.u. intensity for the controls versus $6.45 \pm 0.25 \times 10^5$ a.u. for the shPrickle2 group). Together, these data suggest that the underlying morphological AIS alterations and reduction of sodium channels as a consequence of Prickle2 depletion correlate with changes in AP generation.

We next performed current-clamp recordings from neurons at DIV22 to investigate whether the decrease in excitability at DIV8 was transient or long lasting (Fig. 8I, left). At this stage, the resting membrane potential, as determined from current-clamp recordings, was more hyperpolarized than at DIV8 but not different between the two groups (Table 2). The input resistance remained higher in Prickle2-depleted neurons than in controls. We next assessed AP firing as a response to long current injection steps. Control neurons continuously increased their firing rate with larger current injections, whereas shPrickle2 neurons fired earlier and, after reaching a peak firing rate at an earlier current threshold, showed a decline in AP frequency (Fig. 8, I, right, and J). Quantification of the firing properties showed that neurons transfected with shPrickle2 had a lower firing threshold and a decreased maximum firing rate compared to the control, while their firing gain remained unchanged (Fig. 8, K and L). Our results suggest that down-regulation of Prickle2 exerts a long-lasting effect on changes in excitability, leading to more excitable neurons and compromised sustained firing of AP.

At DIV22, the Prickle2-depleted neurons maintained the multiple-axon phenotype observed at DIV7, with AnkG staining present on supernumerary axons (Fig. 8, M to N″). Labeling with a pan-Na_v antibody showed that Na_v channel recruitment showed a similar trend

Table 2. Summary table of electrophysiological parameters of cultured neurons. Mean values and SEM for cellular parameters and AP firing properties. Control and shPK2-transfected neurons were recorded in whole-cell patch-clamp mode. Experiments were done in triplicate.

Comparison	Means ± SEM	*n*	Test	*P* value
		**DIV8**		
Resting membrane potential	Control: −50.1 ± 2.9 mV	10	Unpaired *t* test	0.5
	shPk2: −52.7 ± 2.5 mV	12		
Input resistance	Control: 722 ± 104 megohms	10	Unpaired *t* test	0.004
	shPk2: 1406 ± 269 megohms	12		
Firing threshold	Control: 25.6 ± 6 pA	10	Unpaired *t* test	0.13
	shPk2: 38 ± 5 pA	10		
Slope *F-I* curve	Control: 0.29 ± 0.04 Hz/pA	10	Unpaired *t* test	0.78
	shPk2: 0.27 ± 0.06 Hz/pA	10		
Maximum firing frequency	Control: 7.4 ± 1.7 Hz	10	Mann-Whitney test	0.035
	shPk2: 3.3 ± 0.9 Hz	10		
		**DIV22**		
Resting membrane potential	Control: −59.9 ± 1 mV	12	Unpaired *t* test	0.4
	shPk2: −58.8 ± 0.8 mV	17		
Input resistance	Control: 183.6 ± 35.8 megohms	12	Mann-Whitney test	0.034
	shPk2: 283.7 ± 35.5 megohms	17		
Firing threshold	Control: 198 ± 23 pA	12	Mann-Whitney test	0.03
	shPk2: 138 ± 22 pA	17		
Slope F-I curve	Control: 62.6 ± 14 Hz/pA	12	Mann-Whitney test	0.8
	shPk2: 99.7 ± 25.6 Hz/pA	17		
Maximum firing frequency	Control: 19.2 ± 3.7 Hz	12	Mann-Whitney test	0.39
	shPk2: 16.0 ± 3.7 Hz	17		
		**DIV8 vs. DIV22**		
Resting membrane potential (all neurons pooled)	DIV8: −51.5 ± 1.9 mV	22	Unpaired *t* test	0.0001
	DIV22: −59.3 ± 0.6 mV	29		

to the presence of AnkG (Fig. 8, O to P‴). Although reduced compared to controls, the levels of AnkG and Na_v appeared higher than at DIV8. Together, our findings revealed that Prickle2 is required to establish the AIS in developing neurons and thereby plays a pivotal role in regulating axonal cytoskeletal organization and neuronal excitability.

DISCUSSION

Our results define Prickle2 as a neuronal polarity determinant and a previously unidentified component of the AIS, participating in its early assembly and stabilization via direct binding to AnkG and MT bundling.

Prickle2 is the earliest binding partner of AnkG480

Our data show that AnkG and Prickle2 colocalize at the earliest stages of neuronal polarity, with a similar spatiotemporal profile of expression. The two proteins are first present in all neurites at the time of axonal specification, followed shortly thereafter by enrichment and stabilization of both proteins at the nascent axon, supporting a role in the initiation of the formation of the AIS. The two proteins appear to be codependent for their stabilization and accumulation at the AIS, as their colocalization was systematic, whether in clusters or vesicles, and the depletion of one led to a complete absence or a severe decrease in AIS enrichment of the other. The distribution of the two proteins in the nascent axon was often fragmented in the most distal region of the axon (see Fig. 2D), a profile that resembles that reported in neurons depleted for the distal axonal components AnkB and β2-spectrin (*14*).

Prickle2 binds all three AnkG isoforms through the LIM domain and the C terminus, including the C2 domain with an apparent stronger affinity for the later. In *Drosophila*, an interaction between the Ankyrin repeats of another core PCP protein, Diego (Diversin or Ankrd6 in mammals), and the C2 domain of Prickle has been described, suggesting a conserved protein-protein interaction motif (*53*). The membrane-binding domain of AnkG, and notably the Ankyrin repeat region, has been shown to bind to various types of ion channels and cell adhesion proteins, probably because of the relative easiness of interaction (*36*). One important consequence of Prickle2 binding to a domain common to the three AnkG isoforms is that Prickle2 probably interacts with, and may modulate, AnkG190 at the spines, as both proteins are also present in this compartment (*31*, *54*). However, this 190 isoform lacks the C-terminal portion

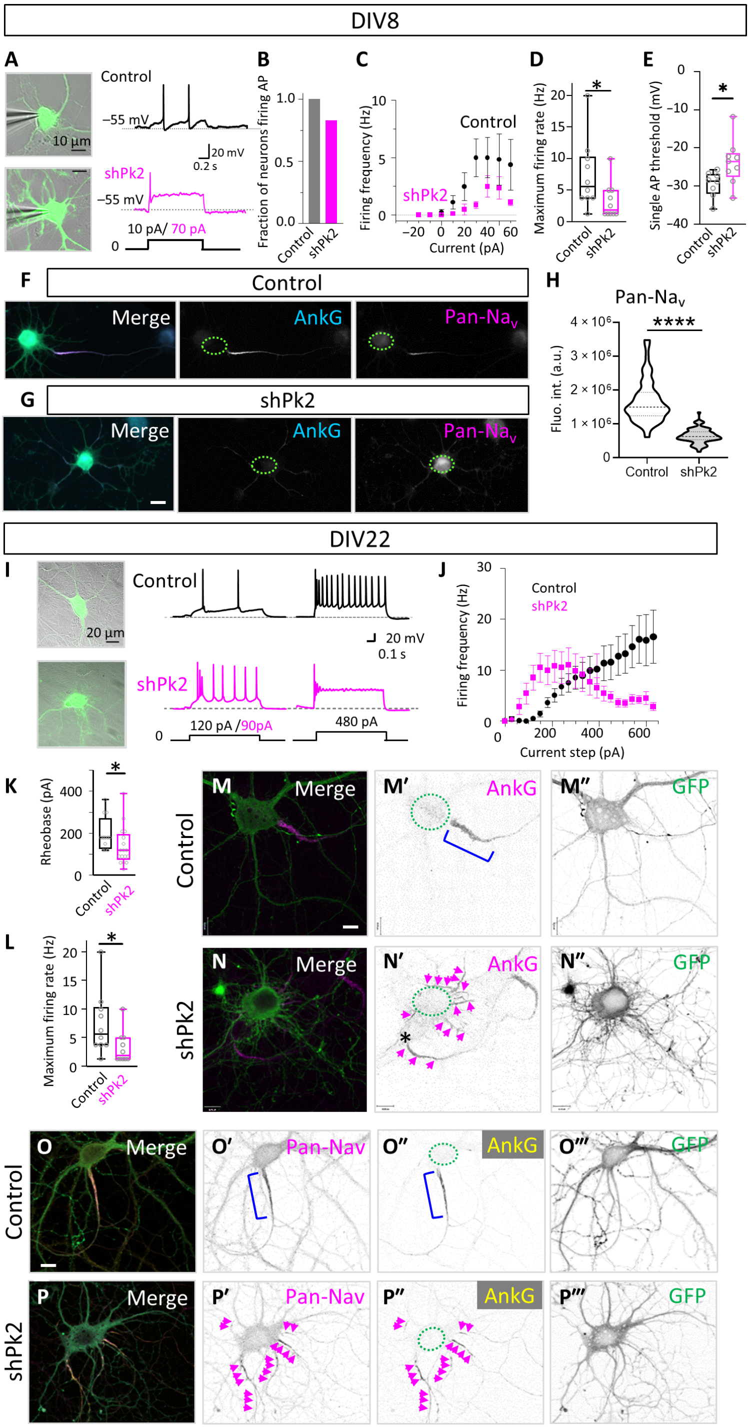

Fig. 8. Pk2 depletion alters the excitability of the neuron. (**A**) Left: Oblique contrast images overlaid with GFP fluorescence for identification of transfected neurons at DIV8. Right: Voltage responses from control and Pk2-depleted neurons to long current injections (bottom). At threshold potential, the depleted neuron requires more current to fire an AP. (**B**) Not all Pk2-depleted neurons could fire AP. (**C**) Firing frequency–current plot determined from 800-ms-long current injections. shPk2 neurons fire less AP. (**D**) The maximal AP firing rate is lower in Pk2-depleted neurons compared to controls. (**E**) Single APs showed that Pk2-depleted neurons had a depolarized AP threshold. (**F** and **G**) Neurons depleted for Pk2 and labeled at DIV7 for Na_V channels. Scale bars, 10 μm. (**H**) Fluorescence intensity levels in controls and shPk2 neurons at DIV7 show strong down-regulations of pan-Na_V. (**I**) Left: Oblique contrast images overlaid with GFP fluorescence at DIV22. Right: Voltage responses from steady-state current injections. (**J**) Firing frequency–current curve at DIV22. (**K**) The rheobase (threshold current) for a single AP is decreased in shPk2 neurons. (**L**) The maximum firing rate is unchanged. (**M** to **N″**) At DIV22, the levels of AnkG are still reduced in Pk2-depleted neurons compared to controls or untransfected neuron [asterisk in (N′)], and the neurons display a multiple-AIS/axon phenotype. (**O** to **P‴**) At DIV22, pan-Na_V immunocytochemistry shows variable levels of Na_V channels on the various AISs that correlate with AnkG labeling. (M to P‴) Bracket indicates the AIS in the control neuron, while magenta arrows point at the different AISs in the Pk2-depleted neuron. Scale bars, 10 μm (A and M to P‴) and 20 μm (I). Statistics, Tables 1 and 2.

encoded by exon 37, preventing MT regulation (*16*), so their interaction will have a distinct, yet unknown, molecular and cellular consequence. The molecular basis of some Prickle2-related neuronal deficits and their relation with pathology such as ASD and epilepsy could therefore be revisited on the basis of these results (*26*, *27*, *31*, *54*).

Prickle2 is a regulator of polarity and AIS formation via AnkG-dependent MT binding

While some Prickle2-depleted neurons had a complete loss of polarity and failed to extend axons, others display supernumerary axons, and others display a unique axon but with a subpar AIS. This gradual severity in phenotype correlated with Prickle2 and AnkG levels (see fig. S6). These results support the hypothesis that Prickle2 levels control AnkG-dependent MT bundling in a dose-dependent way, thereby determining the number of axons, as well as AIS maturation (see fig. S6). Low doses of the MT-stabilizing drug Taxol also leads to supernumerary axons, similar to what we describe in this study (*13*, *45*, *55*). In vivo, we did not observe Prickle2-depleted neurons with multiple axons, which might be a technical issue due to the very low levels of remaining AnkG labeling. We did observe shPrickle2-expressing neurons that were retained in the ventricular zone of IUE brains, which might be explained by a complete loss of neuronal polarity establishment that disrupted the dorsal migration of the neurons (*10*). A role for Prickle in cell polarity has been reported in mammals, with a loss of apicobasal polarity of epiblast cells due in part to the PET/LIM domains of the protein Prickle1 (*56*).

Prickle2-dependent recruitment to MTs via AnkG is a novel observation here, but previous studies in *Drosophila* suggested an indirect link between Prickle and MT, while the molecular basis of this regulation is unclear. Prickle has been suggested to promote and/or stabilize the polarity of axonal MTs in addition to modulating MT growth dynamics in *Drosophila* larval motor neurons (*57*). In addition, in the classic core PCP models of the *Drosophila* wing and abdomen epithelia, Pk is postulated to bias the apical MT plus ends and thus the movement of some core PCP vesicles toward a specific side of the cell (*58*).

This severe polarity phenotype was not observed when AnkG or TRIM46 levels were modulated in the context of normal Prickle2 expression, although the loss of both AnkG and TRIM46 did affect the proximal polarity of axons, notably MT organization (*12*, *16*, *39*, *45*). The presence of Prickle2 in neurons even before axonal specification supports the hypothesis that Prickle2 participates in the early determination of neuronal polarity via axonal specification, whether by binding to AnkG for MT stabilization and/or through another, independent and not mutually exclusive mechanism. Previous studies modulating the levels of Prickle1 or Prickle2 have reported disruption of neurites, with generally scant axonal/dendrite characterization (*26*, *27*, *59*). Last, although we did not explore the role of Prickle2 in polarity maintenance, recent work from Chowdhury and colleagues (*60*) reported that late Prickle2 depletion in DIV11 neurons leads to a large proportion of neurons with no AnkG, suggesting a loss of polarity maintenance.

Even when neurons maintain a single axon in Prickle2-depleted cells, AnkG labeling was always reduced, as were all of the AIS components we evaluated. Therefore, we propose that Prickle2 is necessary for the initial clustering and the maturation of the AIS. Prickle2 may participate in the stabilization of AnkG at the membrane, as was reported for other proteins in *Drosophila* epithelium (*61*). This finding is consistent with the systematic presence of Prickle2 with AnkG during the earliest stages of development and its systematic decrease in Prickle2-depleted neurons. In addition, the decrease in thickness of the AIS in Prickle2-depleted neurons together with the disruption of actin organization and the strong decrease in MAPs and neurofilament levels are consistent with a more global disruption of the cytoskeleton organization. Further analysis, notably at the ultrastructural level, will be needed to confirm these structural alterations (*15*, *39*, *40*).

The formation of uniformly oriented and fasciculated MT bundles is critical for axonal polarity and AIS formation and is presumed to depend on a feedback mechanism involving TRIM46 and AnkG, although no direct interactions have been observed between the proteins (*17*, *39*, *45*, *62*). Our data suggest that Prickle2 binds to AnkG480 to mediate the recruitment of the complex to the MT lattice at the time of axonal specification. Prickle2 therefore promotes MT bundling independent of TRIM46 but in a close interplay with TRIM46-dependent mechanisms to participate in the establishment and stabilization of the AIS (*17*). The presence of an EB-binding site in the tail domain at the C terminus of the AnkG480 isoform is key for driving AIS assembly and forming MT bundles in our assay [(*15*, *16*); this manuscript]. While previous studies suggested that AnkG could serve as an early MT bundler, our data suggest that Prickle2 is necessary for that function (*15*, *40*).

Prickle2 depletion also disrupted actin cytoskeleton periodicity at the AIS. Similar disruptions were observed with inhibitors of actin-associated molecules such as tropomyosin 3.1 (*63*) or down-regulation of β2-spectrin levels (*64*) or even in axonal degeneration contexts (*65*). Currently, the mechanism by which Prickle2 directly or indirectly modulates actin ring formation remains unclear, but it could be indirect as a correlation between actin periodicity and MT distribution has been reported at the axon (*66*).

Neuronal polarization most likely involves sequential interconnected molecular cellular events driving axon specification and AIS formation, dendrite determination, and neuritogenesis. More or less concomitantly, the AIS forms and stabilizes. Prickle2 is most likely involved in these two interconnected events, which confounds the results. We propose that finely controlled spatiotemporal events involving Prickle2 determine its functions in neurons. The complex depletion phenotype is a consequence of the disruption of the availability of Prickle2 in neurons at the time of neuronal polarity and AIS formation. Together with the systematic colocalization of Prickle2 and AnkG in undifferentiated neurites, these results suggest that Prickle2 represents one of the molecular biases tipping a neurite to become an axon. A complete lack of the protein inhibits axon formation (thus AIS formation), a low level of Prickle2 availability strongly affects both events, and a small decrease is not sufficient to affect neuronal polarity but affects AIS formation/stability (see fig. S6). Prickle2 and AnkG appear to be codependent during these events, and some of the defects observed at the AIS in the absence of Prickle2 could be due not only to the secondary decrease in AnkG but also to additional Prickle2-dependent mechanisms. We are currently unable to clarify how Prickle2 participates in these two events, which are inherently connected, or whether they involve the same protein complex.

Prickle2 function in ASD and epilepsy

The observed lower excitability and lack of sodium channel clustering at the AIS of DIV8 Prickle2-depleted neurons are reminiscent of the excitability profile of Purkinje neurons from ANK3 knockout

mice that show deficiencies in AP initiation and a decrease in rapid, repetitive firing as a consequence of an absence of AIS sodium channel clustering (*52*). This electrical profile, however, evolves, and at DIV22, the neurons have increased excitability properties. This result might be due to a lack of effectiveness of the shPrickle2 construct after 3 weeks in vitro, leading to a partial recovery of Prickle2, AnkG, and Na_v channels at the AIS, while maintaining a multiple-axon profile. However, they are also consistent with a disruption of the intrinsic electrical properties of the neuron. The reduction in firing capability during sustained depolarization at DIV22 is consistent with the disruption of the cytoskeleton organization and the fragmented sodium channel clusters. In turn, sodium channel fragmentation/decrease lowers the critical density of sodium channels needed for AP initiation (*4*). This conclusion is not contradictory with the observed higher excitability, as sodium channels in the different clusters can be presumably activated at the same time, leading to a net higher excitability.

Our in vitro results are consistent with a decreased seizure threshold and global increased excitability observed in *Prickle* mutants (*26*). The authors noted that the deletion of only one allele of *Prickle1* or Prickle2 was sufficient for these animals to be more sensitive to developing seizures, further suggesting different contributions of *Prickle1* and Prickle2 to an epileptic phenotype and a dosage-dependent effect of Prickle2 on proper neuronal function.

Recent studies have reported similar hyperexcitable neurons that carry ASD- and epilepsy-related mutations for SCN2A (*Sodium channel protein type 2 subunit alpha*) (*67*, *68*). Both epilepsy and ASD are also reported in patients with *PRICKLE2* mutations (*26*, *27*), while *Prickle2* knockout mice display a lower threshold for seizures (*26*) and ASD-like behaviors (*27*). The latter study was mostly focused on the presence/role of Prickle2 at the spine, while our data further suggest that these pathologies might also have an AIS component. Together, our data and these previously published reports demonstrate that Prickle2 has several molecular and cellular functions in neurons, shaping their polarity and their mature electrical properties.

Overall, we identified Prickle2 as a unique hub for neuronal function and excitability by identifying a new role for this protein at the axon and the AIS that complements its function in dendritic spines. Our results suggest that Prickle2 is a powerful candidate to study neuronal development and function in health and disease. Last, our results establish a relationship between neuronal polarity and PCP, with Prickle2 as a molecular link between the two processes.

MATERIALS AND METHODS

Experimental design

Sample sizes were not predetermined using power analysis, since they were not chosen on the basis of prespecified effect size. Instead, multiple independent experiments were carried out using several biological replicates. Detailed descriptions of sample size and statistical analysis used to test normality and calculate *P* values are given in Tables 1 and 2, in supplementary figure legends, and in the "Data and statistical analysis" section. Data were collected and analyzed by multiple researchers, who were blinded to the conditions whenever experimentally possible.

Animals

All procedures involving animals were done in accordance with the European Union Directives (2010/63/EU) and the University of Bordeaux Ethical Committee and French Research Ministry (APAFIS #26908 and #25075). Rats had free access to food and water and were housed in polypropylene cages under controlled conditions (at 22° ± 2°C, with lights on from 7:00 a.m. to 7:00 p.m., assuring a 12/12-hour light/dark cycle). Sprague-Dawley rats (Janvier Labs, strain RjHan:SD, RRID:RGD_1566457) from both sexes were used between E16 and 10 weeks old at the time of the experiments.

Plasmids

The shRNA was cloned into a pSuper vector (Oligoengine) based on previously published sequences for shPrickle2A and shAnkG (*12*, *41*). shAnkG sequence was obtained from B. Dargent (Marseille, France) and recloned in pSuper vector. ShPrickle2B was generated by F.C. (Aix-Marseille University, France) and cloned afterward into a pSuper vector with the sequence 5′-CTACTTCACG-GAGTATGATTG-3′. AnkG270 and AnkG190 constructs tagged with GFP were provided by V. Bennett (Duke University, USA). The AnkG190-MBD-GFP construct, which encompasses the Ank repeats (amino acids 1 to 840), was generated by deletion mutagenesis using the AnkG190 construct as a template. AnkG480-GFP was obtained from C. Hoogenraad (Utrecht University, The Netherlands), and AnkG480NN-GFP was a gift from A.F. MACF43-RFP was used for live imaging (*69*). pEGFP-C3 was obtained from Clontech, and GFP-Prickle2 was a gift from M. Deans (University of Utah, USA). The plasmid for the human Prickle2 cDNA tagged with three flag sequences in the N terminus was purchased from Addgene (#24645). GST-Prickle2 constructs were generated by cloning different human Prickle2 domains using polymerase chain reaction and inserted into the pGEX-4T-1 (GE Healthcare) to create in-frame fusions with the GST sequence. GST-tagged hPrickle2 (amino acid 1 to 323) contains PET and LIM domains, GST-hPrickle2 (amino acid 323 to 844) contains the C-terminal half of the protein, GST-hPrickle2 (amino acid 127 to 844) contains the LIM domains and the C-terminal half of the protein, GST-hPrickle2 (amino acid 1 to 129) contains the PET domain, GST-hPrickle2 (amino acid 127 to 323) contains the LIM domains, GST-hPrickle2 (amino acid 323 to 585) contains the first half of the C-terminal half of the protein, and GST-hPrickle2 (amino acid 585 to 844) contains the second half of the C-terminal half of the protein including the C2 domain (*70*).

Antibodies

The following antibodies were used in this study: rabbit polyclonal anti-Prickle2 (gift from D. Wu, National Institutes of Health, USA) (*35*). The following were from NeuroMab: mouse monoclonal anti-AnkG (used at 1:2000; clone N106/36, 75-146, RRID:AB_2877524), mouse monoclonal anti–β4-spectrin (used at 1:500; clone N393/76, 75-377, RRID:AB_2877419), and mouse monoclonal anti-pan neurofascin (used at 1:500 for immunostaining on fixed neurons and at 1:200 for live imaging; clone A12/18, 75-172, RRID:AB_2282826). Mouse monoclonal anti-pan Na_v (used at 1:200; Sigma-Aldrich, S8809, RRID:AB_477552) or a cell culture supernatant of human embryonic kidney (HEK) 293T cells transfected with a plasmid (Addgene, #128623, RRID:Addgene_128623) as described in (*71*) (used at 1:10), rabbit polyclonal anti-AnkG (used at 1:5000; Synaptic Systems, 386-003, RRID:AB_2661876), guinea pig polyclonal anti-AnkG (used at 1:2000 for immunoblot; Synaptic Systems, 386-005, RRID:AB_2737033), guinea pig polyclonal anti-TRIM46 (used at 1:500; Synaptic Systems, 377 005, RRID:AB_2721101), chicken polyclonal anti-MAP2 (used at 1:20,000; EnCor Biotech, CPCA-MAP2,

RRID:AB_2138173), mouse anti-neurofilament (used at 1:200; DSHB, NF-M, 2H3, RRID:AB_531793), and rat monoclonal anti–tyrosinated tubulin (used at 1:20,000; Abcam, ab6160, RRID:AB_305328) were used. For tags, we used chicken polyclonal anti-GFP (used at 1:3000 for neurons and at 1:15,000 for COS7; Abcam, ab13970, RRID: AB_300798), mouse monoclonal anti-GFP (used at 1:1000; Sigma-Aldrich, 11814460001, RRID:AB_390913), mouse monoclonal anti-Flag (used at 1:1000; Sigma-Aldrich, F1804, RRID:AB_262044), and rabbit polyclonal anti-GFP (used at 1:1000; Millipore, AB3080P, RRID: AB_2630379). For some of the Western blots, custom-made serum rabbit polyclonal anti-Prickle2 was used at 1:2000.

Secondary antibodies were purchased from Jackson ImmunoResearch and Invitrogen (used all of them in between 1:2000 and 1:3000; Alexa Fluor 488, Alexa Fluor 594, and Alexa Fluor 647). For STORM (Stochastic Optical Reconstruction Microscopy) imaging, actin was detected using Alexa Fluor 647 phalloidin (Thermo Fisher Scientific, A22287). For Western blot, secondary antibodies goat anti-mouse or anti-rabbit coupled to the horseradish peroxidase (HRP; used between 1:10,000 and 1:20,000; from Jackson ImmunoResearch), donkey anti–guinea pig HRP (used at 1:40,000; Jackson ImmunoResearch), and mouse anti-rabbit HRP (used at 1:5000; GE Healthcare, UK) were used.

Immunohistochemistry

Animals were perfused transcardially with phosphate buffer (PB), followed by 4% paraformaldehyde (PFA) in PB, and brains were removed and postfixed in 4% PFA for 2 hours at 4°C. Coronal vibratome sections (40 to 50 μm; VT1000S vibratome, Leica) were permeabilized with 0.25 to 0.3% Triton X-100 in 1× phosphate-buffered saline (PBS) for 1 hour at room temperature (RT) and blocked with 5% bovine serum albumin (BSA) and 5% normal goat serum (NGS) in 1× PBS for 2 hours at RT. Primary antibodies were diluted in 1× PBS and incubated overnight at 4°C. Fluorescent secondary antibodies were diluted in 1× PBS or 2% NGS–1× PBS and incubated for 1 hour at RT. Slices were mounted using ProLong Gold antifade medium (Life Technologies) or Fluoromount-G (Electron Microscopy Sciences). Rat brains electroporated in utero were dissected at P5 or P6 and immersed in 4% PFA overnight at 4°C, followed by 24 to 48 hours of incubation with 30% sucrose PBS at 4°C. Brains were sliced on coronal 50-μm sections and processed for immunohistochemistry. For IUE, two pregnant rats were used per surgery and, between three and six GFP-positive brains per rat, were processed for immunostaining.

Neuronal cell culture, transfection, and immunofluorescence

All of the experiments used either 15- or 18-mm coverslips coated with poly-L-lysine (PLL) or laminin: Coverslips were incubated for 2 hours at 37°C with PLL (30 μg/ml) in borax buffer, rinsed with Braun water, and then incubated with laminin diluted in water at 2 μg/ml overnight at 37°C. Coverslips were rinsed the day after with Braun water and filled with Neurobasal medium (Gibco) until the moment of plating.

Sprague-Dawley rats at E18.5 were used for mixed cultures of hippocampal neurons. Hippocampi were collected together in Hanks' balanced salt solution (HBSS; Gibco)/Hepes (Gibco)/penicillin-streptomycin (Gibco) mix and rinsed with cold chopping solution [HBSS/Hepes/deoxyribonuclease (DNase); DNase from Sigma-Aldrich]. To achieve chemical dissociation, a mix of warm chopping solution with trypsin (Gibco) was added to the hippocampi and incubated at 37°C for 12 to 15 min with eventual agitation. After stopping the trypsin with multiple rinses with chopping solution, mechanical dissociation was achieved by gently moving up and down the mix using a Pasteur pipette. Cells were counted and seeded in plating medium containing Neurobasal medium (Gibco), B27 supplement (50×; Gibco), and 2% of fetal bovine serum (FBS). Four hours after plating, plating medium was replaced by conditioned medium obtained from astrocyte cultures. Neurons were kept in a humidified incubator at 37°C and with 5% CO_2.

Neurons were transfected before plating using an Amaxa Rat Neuron Nucleofector kit (Lonza), following the manufacturer's instructions. Nucleofections for shRNA-Prickle2A and shRNA-Prickle2B used 500,000 cells for nucleofection, mixed together with 250,000 of nontransfected neurons to promote survival. Each of these had a control plasmid (pSuper for shPrickle2A and shPrickle2B) used at the corresponding quantities. For live imaging and MT dynamics experiments, neuronal cultures were performed as follows: Primary hippocampal neuron cultures were prepared from E18 rat brains, and 400,000 cells per condition were nucleofected with 3 μg of DNA (2.5 μg of pSUPER-GFP and 0.5 μg of MACF43-FRP) (*49*) using the Amaxa Rat Neuron Nucleofector kit (Lonza) according to the manufacturer's instructions. Cells were plated on coverslips coated with PLL (37.5 μg/ml) and laminin (1.25 μg/ml) at a density of 200,000 per well. Neurons were cultured in Neurobasal medium supplemented with 2% B27 (Gibco), 0.5 mM glutamine (Gibco), 15.6 μM glutamate (Sigma-Aldrich), and 1% penicillin-streptomycin (Gibco) at 37°C in 5% CO_2 and imaged at DIV7 to DIV8.

For immunocytochemistry, neurons were fixed and labeled at defined time points for 10 min with 10% sucrose and 4% PFA at RT. For experiments involving detergent extraction, Triton X-100 was added to a concentration of 0.25% in the fixation mix. Neurons were then permeabilized and blocked with 5% BSA and 0.3% Triton X-100 in PBS for 1 hour at RT, followed by primary antibody incubation for 1 hour at RT. After rinsing, Alexa Fluor secondary antibodies (Jackson ImmunoResearch) were incubated for 30 min at RT, and coverslips were mounted using ProLong Gold antifade medium (Life Technologies). For neurofascin live imaging, neurons were incubated for 5 min with CF640R-coupled (Mix-n-Stain, Biotium) extracellular anti–pan-neurofascin antibody (diluted in Neurobasal) at 37°C. The coverslips were then washed two times with warm Neurobasal, and the original medium was added back before starting imaging. For STORM experiments, neurons were postfixed after rinsing the secondary antibodies with 10% sucrose and 4% PFA for 10 min at RT, followed by three rinses in PBS.

Astrocyte-conditioned medium

The day before the culture, 75-cm^2 plastic dishes were coated with PLL (10 μg/ml) for 2 hours at 37°C and rinsed with Braun water. Three cortices of E18.5 Sprague-Dawley rats were collected together in HBSS (Gibco)/Hepes (Thermo Fisher Scientific) mix and rinsed with cold chopping solution (HBSS/Hepes/DNase; DNase from Sigma-Aldrich). Warm chopping solution with trypsin (Thermo Fisher Scientific) was added to the cortices and incubated at 37°C for 10 to 15 min with eventual agitation. After stopping the trypsin with blocking solution (HBSS/Hepes from Gibco), mechanical dissociation was achieved by gently moving up and down the mix with a Pasteur pipette. Cells were centrifuged for 10 min at 1000 rpm at RT, and the pellet was taken back into the plating medium containing Neurobasal medium (Gibco) and B27 supplement (50×; Gibco).

Four hours after plating, plating medium was replaced by conditioned medium obtained from astrocyte cultures. Neurons were kept in a humidified incubator at 37°C and with 5% CO_2.

Live-cell imaging and MT severing—Photoablation

CF640R-coupled (Mix-n-Stain, Biotium) extracellular anti-pan neurofascin antibody (used 1/200 in Neurobasal; NeuroMab, clone A12/18, 75-172, RRID:AB_2282826) was incubated on live neurons for 5 min at RT. After two washes in Neurobasal, neurons were returned to their conditioned medium until imaged. Live-cell imaging experiments were performed in a Nikon Eclipse Ti-E inverted microscope (Nikon), equipped with a Plan Apo VC 100× 1.40 numerical aperture (NA) oil, a Yokogawa CSU-X1-A1 spinning disk confocal unit (Roper Scientific), a Photometrics Evolve 512 EMCCD (Electron Multiplying Charge Coupled Device) camera (Roper Scientific), and an incubation chamber (Tokai Hit) mounted on a motorized XYZ stage (Applied Scientific Instrumentation), which were all controlled using MetaMorph (Molecular Devices) software. Coverslips were mounted in metal rings and imaged using an incubation chamber that maintains temperature and CO_2 optimal for the cells (37°C and 5% CO_2). Neuron live imaging was performed in full conditioned medium. Time-lapse live-cell imaging of MACF43-RFP was performed with a time acquisition of 1 s. Teem Photonics 355-nm Q-switched pulsed laser was used to perform laser-induced severing and study MT orientation in neurons as described previously (*50*). The severing sites were targeted within 40 μm from the cell body of DIV7 to DIV8 neurites. Axons were identified by their highest fluorescent signal of neurofascin and/or by the relative length of neurites using GFP fluorescence. No sign of toxicity to cells was observed during laser-induced severing.

Image acquisition and processing

Most of the IUE brain slice imaging was performed using a Zeiss AxioImager Z1 (63×/1.40 NA oil objective) equipped with an AxioCam MRm, the Zen software (Zeiss), and a light-emitting diode (LED) light source, Colibri 7, from Zeiss [wavelengths: ultraviolet (UV), 385/30 nm; V, 423/44 nm; B, 469/38 nm; C, 511/44 nm; G, 555/30 nm; Y, 590/27 nm; R, 631/33 nm]. Stacks of 18.98 μm (73 slides, step size of 0.26 μm) were taken for analysis. For dissociated neuron analysis, except for those in Fig. 2, Z-stacks were collected per cell with the abovementioned Zeiss microscope (five slides with a step size of 0.25 μm and a total depth of 1 μm). COS7 cells were imaged as single plane acquisitions. All the acquisitions were collected with sequential wavelength acquisition. A confocal/STED microscope (TCS SP8; Leica) with module STED x3 was used for some of the neurons (DIV1 to DIV3 in Fig. 2), COS7, and tissue acquisitions. For illustration, brains from rat IUE were imaged using a Z step from 0.25. For MT dynamics analysis, movies and images were processed using Fiji (https://imagej.net/Fiji). Kymographs were generated using the ImageJ plugin KymoResliceWide v.0.4 (https://github.com/ekatrukha/KymoResliceWide). Image editing was performed using ImageJ or Photoshop 7.0 (Adobe).

AIS fluorescence and thickness quantifications

For AIS protein level quantifications in control and shRNA-Prickle2A and shRNA-Prickle2B, stacks were processed for maximum intensity projections, and total AIS intensity was calculated as the sum of the intensity of each pixel along the AIS, whose measurement line was 20 pixels wide. We define multiple-AIS/axon phenotype as at least three axons and above based on any positive AIS clustering of AnkG labeling. In multiple-AIS neurons, the intensity values per AIS were averaged to have a single value per neuron. An integrated intensity approach was favored to the mean intensity because of the fragmented profile of the fluorescence under down-regulation conditions. Automation of this analysis was possible thanks to a macro for ImageJ written by F. Cordelieres (Bordeaux Imaging Centre). The same macro was used to draw the linescans of Prickle2 and AnkG profiles at the AIS in cultured neurons at different stages. Linescans in STED images were performed using ImageJ by drawing the lines at the specified regions and exporting the data of the fluorescence profiles. For AIS thickness measurements, stacks (five slides with a step size of 0.25 μm and a total depth of 1 μm) were collected with Zeiss AxioImager Z1 (63×/1.40 NA oil objective) equipped with an AxioCam MRm, the Zen software (Zeiss), and an LED light source, Colibri 7, from Zeiss (wavelengths: UV, 385/30 nm; V, 423/44 nm; B, 469/38 nm; C, 511/44 nm; G, 555/30 nm; Y, 590/27 nm; R, 631/33 nm). Three vertical lines (one for the proximal region and two for the distal part) were traced with ImageJ crossing the AIS from top to bottom along its thickness. The resulting length of the lines (and therefore thickness) was calculated, and the definite number results from the direct measurement for the proximal region and from the average between the two last measurements for the distal part. When the neuron had several AISs, the subsequent number was the average of each of the values from the different AISs. Measurements were done using the green (GFP) channel. For AIS fluorescence intensity measurements in IUE experiments, stacks were processed for maximum intensity projections, and mean intensity along the AIS was measured using ImageJ with a 5-pixel-wide line.

In utero electroporations

E16.5 Sprague-Dawley embryos were injected and electroporated in utero. Timed-pregnant E16.5 females were anesthetized with isoflurane after a preoperative dose of buprenorphine (0.05 mg/kg), 15 min before surgery. The abdomen was cut, and the uterine horn was exposed. One microliter of DNA (0.5 μg/μl for shRNA Prickle2 and 0.25 μg/μl for control plasmid) prepared in endo-free water mixed with 1% Fast-Green dye (Sigma-Aldrich) was injected through the uterine wall into the lateral ventricle of each embryo using pulled glass capillaries (Harvard Apparatus). Capillaries were prepared using a needle pipette puller (Narishige, PC-100), and injection was performed with a Femtojet injector (Eppendorf). For electroporation, five 50-ms pulses at 55 V with 950-ms breaks were delivered through the embryonic brain to target the cortex using 7-mm electrode paddles connected to a BTX ECM830 electroporator (Harvard Apparatus). After the procedure, the abdomen was filled with a 37°C-warmed PBS solution, and the wound was closed using surgical suture. Animals were put back in their home cage in the animal facility. Rats gave birth at E22.5, and the pups were kept with their mother until collection at P5 or P6.

STORM imaging

STORM acquisitions were performed at the Bordeaux Imaging Center. Coverslips (18 mm) were incubated with fluorescent beads (Invitrogen) diluted in 1× PBS at 1/1000 for 5 min at RT, washed thrice with 1× PBS, and then placed in a Ludin chamber. STORM media consisted of 50 mM tris (pH 8), 10 mM NaCl, 10% glucose, 100 mM Cysteamine (MEA), pyranose oxidase (3.5 U/ml), and

catalase (40 μg/ml). The STORM microscope was a Nikon Ti Eclipse (Nikon France S.A.S., Champigny-sur-Marne, France) equipped with a Perfect Focus System, a motorized stage TI-S-ER, and an azimuthal Ilas^2 TIRF (total internal reflection fluorescence) arm (Gataca Systems, Massy, France) coupled to a laser bench containing 405-nm (100-mW), 491-nm (150-mW), 532-nm (1-W), 561-nm (200-mW), and 642-nm (1-W) diodes. Images were acquired using objective Apo TIRF 100× 1.49 NA oil and a sensitive Evolve EMCCD camera (Photometrics, Tucson, USA). The AIS of control and shPrickle2 neurons was identified in TIRF settings thanks to AnkG staining (staining specifics in the "Antibodies" and "Neuronal cell culture, transfection, and immunofluorescence" sections). Phalloidin was then imaged at the AIS on STORM settings by using the 642-nm laser at 1000 mW for single 647-channel acquisitions. Between 50,000 and 100,000 frames were taken per neuron. PalmTracer software was used for image treatment and reconstruction (developed by J. Baptiste Sibarita's team at the Interdisciplinary Institute of Neurosciences, Bordeaux). The periodicity of actin rings was determined by drawing a line through the actin immunosignal and measuring the pixel gray value intensity along this line. Absolute peak locations were determined by using an automated peak detection mechanism (AxographX). The distance between peaks was subsequently calculated as the differences between two peaks. We calculated histograms for control and shPrickle2-transfected neurons and fitted the data with a Gaussian equation.

HEK293T and COS-7 culture, transfection, and immunofluorescence

HEK293 containing the SV40 large T antigen [HEK293T, from the American Type Culture Collection (ATCC), CRL-11268-G1TM, RRID:CVCL_0063] and COS-7 (ATCC, CRL-1651, RRID:CVCL_0224) were cultured with advanced Dulbecco's modified Eagle's medium (DMEM; Gibco) supplemented with 10% FBS (Gibco), 1% L-glutamine (200 mM; Gibco), and 1% penicillin-streptomycin (10,000 U/ml; Gibco). Cell cultures were grown in a humidified incubator at 37°C with 5% CO_2. Subculture procedure was done following the indications of the company. For transfecting HEK293T, cells were plated onto 10-cm dishes in complete DMEM at a suitable dilution to reach 30 to 40% confluence before transfection. Transfection was performed with polyethylenimine (PEI; 1 mg/ml; linear, Polysciences Inc.) with a ratio of 2.5 μl:1 μg of cDNA for 16 μg of AnkG cDNA and of 3.5 μl:1 μg of cDNA for 4 μg of Prickle2 cDNA. Plasmid constructs were mixed with 400 μl of serum-free DMEM before addition of PEI, vortexed, and incubated for 15 min at RT. After that, 400 μl of the mix was added dropwise to each dish.

For transfecting COS-7, cells were plated onto six-well plates with three coverslips (15 mm) per well in complete DMEM at a suitable dilution to reach 30 to 40% confluence before transfection. Cells were transfected using calcium phosphate (*72*). The different cDNAs were prepared with 12.5 μl of $CaCl_2$ and H_2O until a total volume of 100 μl. For cDNA quantities, Flag-Prickle2 was used at 2 μg for single transfection and at 1, 2, and 3 μg in combination with AnkG480-GFP, which was used at 2 μg at all times. AnkG190-GFP and AnkG480NN-GFP were also used at 2 μg. The resulting mix was applied dropwise to 100 μl of 2× HBS incubated for 30 min at RT in the dark and applied to the cells. The plates were then placed back in the incubator for 5 hours. After that, three washes with serum-free DMEM were performed, and the cells were placed in the incubator for 10 min. Last, serum-free DMEM was replaced by complete medium. Twenty-four hours later, cells were fixed and processed for immunocytochemistry.

COS-7 cells were fixed with 4% sucrose and 4% PFA for 10 min at RT and permeabilized for 10 min at RT with 0.25% Triton X-100–1× PBS. A blocking step using 5% BSA (Euromedex) and 0.05% Tween 20 (Sigma-Aldrich)–1× PBS was performed for 30 min at RT. Primary antibodies were diluted in 1× PBS and incubated at RT for 1 hour. Secondary antibodies were mixed in 1% BSA–1× PBS and incubated for 45 min at RT. Coverslips were mounted using Fluoromount-G antifade medium (Electron Microscopy Sciences) or ProLong Gold (Life Technologies).

GST production and purification

Different GST-tagged PRICKLE2 constructs were purified from *Escherichia coli* competent strain BL21 (Agilent Technologies) supernatants by standard affinity purification on glutathione-Sepharose 4B beads (GE Healthcare, UK). One colony of transformed bacteria per construct was amplified in 200 ml of LB medium (ampicillin resistance) during 4 hours at 37°C. When optical density at 560 nm reached 1, isopropyl-β-D-thiogalactopyranoside at 0.1 mM was added to induce plasmid expression (4 hours). After centrifugation at 3500*g*, pellets were flash-frozen in dry ice and resuspended in tris-buffered saline (TBS) supplemented with lysozyme (200 μg/ml; 30 min on ice). After sonication, 15 mM dithiothreitol, 10 mM EDTA, 1 mM sodium fluoride, 1 mM sodium orthovanadate, a protease inhibitor tablet (Roche), and 1.5% sodium lauroyl sarcosinate (sarkosyl) were added to the samples (15 min on ice). Samples were then centrifuged at 186,000*g* for 40 min before adding 4% Triton X-100 to supernatants to stop the action of sarkosyl. Prewashed glutathione Sepharose beads were incubated with supernatants (2 hours at 4°C) to link GST-tagged proteins, and after an overnight wash with TBS, beads were resuspended in TBS and kept at 4°C for later use. To evaluate purified GST amounts, samples were loaded on SDS–polyacrylamide gel electrophoresis (PAGE) and ran at 150 V for 1 hour, and acrylamide/bis gel was stained with Coomassie Brilliant Blue [45% methanol, 10% glacial acetic acid, and Coomassie Brilliant Blue R250 (3 g/liter; Pierce) in distilled water].

IP and pulldown assays

Forty-eight hours after transfection, HEK293T cells were collected in cold PBS and centrifuged at 500*g* for 10 min (4°C). Pellets were flash-frozen in dry ice and resuspended in lysis buffer [50 mM tris-HCl (pH 7.5), 150 mM NaCl, 2 mM EDTA, and a protease inhibitor tablet (cOmplete, Pierce)]. Cells were then homogenized with a polytron and sonicated. For solubilization, 1% Triton X-100 was added to samples (2 hours at 4°C), and solubilized material was centrifuged at 112,000*g* for 40 min. Proteins in supernatant were then immunoprecipitated with 8 μg of rabbit anti-GFP or 8 μg of rabbit immunoglobulin G (IgG; Thermo Fisher Scientific) for 2 hours at 4°C. Protein A agarose beads (50 μg; Pierce), prerinsed with PBS and 0.1% Triton X-100, were incubated with samples overnight at 4°C. After rinsing the beads with increasing amounts of NaCl (50 to 150 mM), proteins were collected in 2× sample buffer [125 mM tris (pH 6.8), 20% glycerol, 1% bromophenol blue, 4% SDS, and 10% β-mercaptoethanol], and bound complexes were analyzed by SDS-PAGE and Western blotting.

For endogenous IP, 250 to 300 mg of cortices from P21 rats were collected in lysis buffer [50 mM tris (pH 7.5), 150 mM NaCl, 2 mM EDTA, and protein inhibitor cocktail (cOmplete ULTRA Tablets,

Roche)] and homogenized with a polytron. Proteins were solubilized with 1% Triton X-100 during 3 hours at 4°C before centrifugation at 112,000*g* for 40 min. Eight micrograms of guinea pig anti-AnkG or 8 μg of guinea pig IgG was added to supernatant (3 hours at 4°C) before incubation overnight at 4°C with 50 μl of protein A/G agarose beads (Pierce) prerinsed four times with PBS and 0.1% Triton X-100. Washing of beads and protein recovery were done in the same manner as for in vitro IP.

A pulldown assay was performed with AnkG270-GFP in HEK293T cells. Cell pellet was resuspended, homogenized, and sonicated in tris-HCl (pH 7.5) containing 5 mM EDTA, 150 mM NaCl, 1 mM sodium fluoride, 1 mM sodium orthovanadate, and one tablet of protease inhibitors. Solubilization was achieved with 1% Triton X-100 for 1 hour and 30 min at 4°C and with 0.5% SDS for 30 min at 4°C. Solubilized material was centrifuged at 150,000*g* for 40 min, and supernatant was incubated overnight with the beads attached to the GST alone or to the GST-Prickle2 fusion proteins. After four washes with TBS and 0.1% Triton X-100, the beads were suspended in 2× sample buffer and subjected to SDS-PAGE and Western blotting. GST amounts per pulldown were evaluated by staining the membrane in Ponceau solution [0.2% (w/v) Ponceau S in 3% (v/v) acetic acid].

Electrophysiology recordings and data analysis

Neuronal cultures from DIV7 or DIV8 to DIV21 or DIV22 were transferred to RT recording solution composed of 125 mM NaCl, 3 mM KCl, 25 mM Hepes, 2 mM $CaCl_2$, 1 mM $MgSO_4$, and 10 mM glucose, and the pH was set to 7.4 with NaOH. All chemicals were purchased from Sigma-Aldrich (Saint-Quentin-Fallavier, France). For electrophysiology recordings, neurons were continuously perfused with recording solution, and recordings were carried out at RT to extend the survival of the neurons. Nucleofected neurons were identified by their GFP fluorescence on upright microscopes [LN-scope (Luigs-Neumann, Ratingen, Germany) or Olympus BX51 (Olympus, Rungis Cedex, France)] using a standard GFP filter set (GFP-30LP-B-000, Semrock, IDEX Health & Science LLC, Rochester, NY, USA) and LED illumination for transmitted and epifluorescence light. Fluorescence exposure was kept as short as possible and was limited with a field stop in the excitation light path. Neurons exhibiting very strong fluorescence were not selected for experiments. Initial experiments were performed blinded for transfection conditions, but morphological alterations were notable; thus, later experiments were performed unblinded. Intracellular solution for whole-cell recordings was composed of 130 mM K-gluconate, 10 mM KCl, 10 mM Hepes, 4 mM Mg–adenosine 5′-triphosphate, 0.3 mM Na_2–guanosine 5′-triphosphate, and 10 mM Na_2-phosphocreatine, and the pH was set to 7.25 with KOH and supplemented with biocytin (5 mg/ml). When filled with intracellular solution, borosilicate glass pipettes (Sutter Instruments, Novato, CA, USA, BF150-75-10) had a resistance of, on average, 6.4 megohms. Whole-cell current- and voltage-clamp recordings were performed with Sutter double IPA amplifiers (Sutter Instruments) controlled by SutterPatch software (Sutter Instruments). Recordings were sampled with a minimum rate of 10 kHz and four-pole Bessel filtered at 5 kHz. In current-clamp recordings, bridge balance and capacitance were compensated. At DIV22, neurons with a resting membrane potential more depolarized than −50 mV were excluded from analysis.

All recordings were analyzed with SutterPatch software and Igor Pro (Wavemetrics, Lake Oswego, OR, USA) or AxographX (Axograph.com). Analysis routines were set up in SutterPatch to automatize analysis. Input resistance was determined from a linear fit to the steady-state voltage in response to small current injection steps (±5 pA at DIV8 and ±30 pA at DIV22). APs were automatically detected with the built-in detection mechanism of SutterPatch, and the inclusion criteria for an AP were defined when the voltage crossed the threshold of 0 mV. Resting membrane potential was determined in current clamp during zero-current injection.

Polarity index

This analysis was done following (*44*). Several lines were traced along the dendrites and axons from control and shPrickle2 neurons. Mean intensities in these segments for AnkG were calculated using ImageJ. Intensity values from several dendrites or axons were averaged per neuron to calculate the PI. The following formula was used: PI = $(I_d - I_a)/(I_d + I_a)$, where Id represents the intensity of the dendrite and Ia represents the intensity of the axon. PI was calculated per neuron. A PI of 0 represents a nonpolarized distribution of the protein, whereas PI > 0 or PI < 0 indicates polarization toward dendrites or axons, respectively.

Data and statistical analysis

Experimenters were blinded to the conditions to perform the data analysis. Data analysis and plotting were performed using GraphPad (San Diego, CA). Details about data display in graphics, sample size, and statistical tests are found Tables 1 and 2. To choose the right statistical test, datasets were first tested for normality using D'Agostino and Pearson or Shapiro-Wilk tests. Statistical significance was then determined by the corresponding test, considering the distribution of the data and the variance (equal or unequal). We generally refer to biological replicates as independent experiments. For IUE experiments, we considered the number of brains as biological replicates, while the independent experiments refer to the pregnant rats. Statistical significance was defined as $*P < 0.05$, $**P < 0.01$, $***P < 0.001$, and $****P < 0.0001$, and ns, not significant.

SUPPLEMENTARY MATERIALS

Supplementary material for this article is available at https://science.org/doi/10.1126/sciadv.abo6333

View/request a protocol for this paper from *Bio-protocol*.

REFERENCES AND NOTES

1. M. N. Rasband, The axon initial segment and the maintenance of neuronal polarity. *Nat. Rev. Neurosci.* **11**, 552–562 (2010).
2. M. S. Grubb, J. Burrone, Building and maintaining the axon initial segment. *Curr. Opin. Neurobiol.* **20**, 481–488 (2010).
3. S. L. Jones, T. M. Svitkina, Axon initial segment cytoskeleton: Architecture, development, and role in neuron polarity. *Neural Plast.* **2016**, e6808293 (2016).
4. M. H. P. Kole, S. U. Ilschner, B. M. Kampa, S. R. Williams, P. C. Ruben, G. J. Stuart, Action potential generation requires a high sodium channel density in the axon initial segment. *Nat. Neurosci.* **11**, 178–186 (2008).
5. A. Foust, M. Popovic, D. Zecevic, D. A. McCormick, Action potentials initiate in the axon initial segment and propagate through axon collaterals reliably in cerebellar Purkinje neurons. *J. Neurosci.* **30**, 6891–6902 (2010).
6. S. L. Palay, C. Sotelo, A. Peters, P. M. Orkand, The axon hillock and the initial segment. *J. Cell Biol.* **38**, 193–201 (1968).
7. K. Xu, G. Zhong, X. Zhuang, Actin, spectrin, and associated proteins form a periodic cytoskeletal structure in axons. *Science* **339**, 452–456 (2013).
8. C. Leterrier, The axon initial segment: An updated viewpoint. *J. Neurosci.* **38**, 2135–2145 (2018).
9. A. Prokop, Cytoskeletal organization of axons in vertebrates and invertebrates. *J. Cell Biol.* **219**, e201912081 (2020).

10. J. Hakanen, N. Ruiz-Reig, F. Tissir, Linking cell polarity to cortical development and malformations. *Front. Cell. Neurosci.* **13**, 244 (2019).
11. W. Guo, K. Stoklund Dittlau, L. Van Den Bosch, Axonal transport defects and neurodegeneration: Molecular mechanisms and therapeutic implications. *Semin. Cell Dev. Biol.* **99**, 133–150 (2020).
12. K. L. Hedstrom, X. Xu, Y. Ogawa, R. Frischknecht, C. I. Seidenbecher, P. Shrager, M. N. Rasband, Neurofascin assembles a specialized extracellular matrix at the axon initial segment. *J. Cell Biol.* **178**, 875–886 (2007).
13. H. Witte, D. Neukirchen, F. Bradke, Microtubule stabilization specifies initial neuronal polarization. *J. Cell Biol.* **180**, 619–632 (2008).
14. M. R. Galiano, S. Jha, T. S.-Y. Ho, C. Zhang, Y. Ogawa, K.-J. Chang, M. C. Stankewich, P. J. Mohler, M. N. Rasband, A distal axonal cytoskeleton forms an intra-axonal boundary that controls axon initial segment assembly. *Cell* **149**, 1125–1139 (2012).
15. P. M. Jenkins, N. Kim, S. L. Jones, W. C. Tseng, T. M. Svitkina, H. H. Yin, V. Bennett, Giant ankyrin-G: A critical innovation in vertebrate evolution of fast and integrated neuronal signaling. *Proc. Natl. Acad. Sci. U.S.A.* **112**, 957–964 (2015).
16. A. Fréal, C. Fassier, B. Le Bras, E. Bullier, S. De Gois, J. Hazan, C. C. Hoogenraad, F. Couraud, Cooperative Interactions between 480 kDa Ankyrin-G and EB proteins assemble the axon initial segment. *J. Neurosci.* **36**, 4421–4433 (2016).
17. A. Fréal, D. Rai, R. P. Tas, X. Pan, E. A. Katrukha, D. van de Willige, R. Stucchi, A. Aher, C. Yang, A. F. M. Altelaar, K. Vocking, J. A. Post, M. Harterink, L. C. Kapitein, A. Akhmanova, C. C. Hoogenraad, Feedback-driven assembly of the axon initial segment. *Neuron* **104**, 305–321.e8 (2019).
18. A. Ghosh, E. L. Malavasi, D. L. Sherman, P. J. Brophy, Neurofascin and Kv7.3 are delivered to somatic and axon terminal surface membranes en route to the axon initial segment. *ELife* **9**, e60619 (2020).
19. M. Montcouquiol, M. W. Kelley, Development and patterning of the cochlea: From convergent extension to planar polarity. *C.S.H. Perspect. Med.* **10**, a033266 (2020).
20. E. Bazellières, V. Aksenova, M. Barthélémy-Requin, D. Massey-Harroche, A. Le Bivic, Role of the crumbs proteins in ciliogenesis, cell migration and actin organization. *Semin. Cell Dev. Biol.* **81**, 13–20 (2018).
21. B. Margolis, The Crumbs3 polarity protein. *C.S.H. Perspect. Biol.* **10**, a027961 (2018).
22. E. Rodriguez-Boulan, I. G. Macara, Organization and execution of the epithelial polarity programme. *Nat. Rev. Mol. Cell. Biol.* **15**, 225–242 (2014).
23. M. Montcouquiol, R. A. Rachel, P. J. Lanford, N. G. Copeland, N. A. Jenkins, M. W. Kelley, Identification of Vangl2 and Scrb1 as planar polarity genes in mammals. *Nature* **423**, 173–177 (2003).
24. R. Hale, D. Strutt, Conservation of planar polarity pathway function across the animal kingdom. *Annu. Rev. Genet.* **49**, 529–551 (2015).
25. N. Sans, J. Ezan, M. M. Moreau, M. Montcouquiol, Chapter 13—Planar cell polarity gene mutations in autism spectrum disorder, intellectual disabilities, and related deletion/duplication syndromes, in *Neuronal and Synaptic Dysfunction in Autism Spectrum Disorder and Intellectual Disability*, C. Sala, C. Verpelli, Eds. (Academic Press, 2016), pp. 189–219.
26. H. Tao, J. R. Manak, L. Sowers, X. Mei, H. Kiyonari, T. Abe, N. S. Dahdaleh, T. Yang, S. Wu, S. Chen, M. H. Fox, C. Gurnett, T. Montine, T. Bird, L. G. Shaffer, J. A. Rosenfeld, J. McConnell, S. Madan-Khetarpal, E. Berry-Kravis, H. Griesbach, R. P. Saneto, M. P. Scott, D. Antic, J. Reed, R. Boland, S. N. Ehaideb, H. El-Shanti, V. B. Mahajan, P. J. Ferguson, J. D. Axelrod, A. E. Lehesjoki, B. Fritzsch, D. C. Slusarski, J. Wemmie, N. Ueno, A. G. Bassuk, Mutations in Prickle orthologs cause seizures in flies, mice, and humans. *Am. J. Hum. Genet.* **88**, 138–149 (2011).
27. L. P. Sowers, L. Loo, Y. Wu, E. Campbell, J. D. Ulrich, S. Wu, L. Paemka, T. Wassink, K. Meyer, X. Bing, H. El-Shanti, Y. M. Usachev, N. Ueno, R. J. Manak, A. J. Shepherd, P. J. Ferguson, B. W. Darbro, G. B. Richerson, D. P. Mohapatra, J. A. Wemmie, A. G. Bassuk, Disruption of the non-canonical Wnt gene PRICKLE2 leads to autism-like behaviors with evidence for hippocampal synaptic dysfunction. *Mol. Psychiatry* **18**, 1077–1089 (2013).
28. C. Harrison, H. Shao, H. Strutt, D. Strutt, Molecular mechanisms mediating asymmetric subcellular localisation of the core planar polarity pathway proteins. *Biochem. Soc. Trans.* **48**, 1297–1308 (2020).
29. P. N. Adler, Chapter 1—The frizzled/stan pathway and planar cell polarity in the Drosophila wing, in *Current Topics in Developmental Biology*, Y. Yang, Ed. (Academic Press, 2012), vol. 101, pp. 1–31.
30. M. Mlodzik, Planar cell polarity: Moving from single cells to tissue-scale biology. *Development* **147**, dev186346 (2020).
31. Y. Hida, M. Fukaya, A. Hagiwara, M. Deguchi-Tawarada, T. Yoshioka, I. Kitajima, E. Inoue, M. Watanabe, T. Ohtsuka, Prickle2 is localized in the postsynaptic density and interacts with PSD-95 and NMDA receptors in the brain. *J. Biochem.* **149**, 693–700 (2011).
32. Y. Ban, T. Yu, B. Feng, C. Lorenz, X. Wang, C. Baker, Y. Zou, Prickle promotes the formation and maintenance of glutamatergic synapses by stabilizing the intercellular planar cell polarity complex. *Sci. Adv.* **7**, eabh2974 (2021).
33. J. Casal, B. Ibáñez-Jiménez, P. A. Lawrence, Planar cell polarity: The prickle gene acts independently on both the Ds/Ft and the Stan/Fz systems. *Development* **145**, dev168112 (2018).
34. O. M. Mapp, G. S. Walsh, C. B. Moens, M. Tada, V. E. Prince, Zebrafish Prickle1b mediates facial branchiomotor neuron migration via a farnesylation-dependent nuclear activity. *Development* **138**, 2121–2132 (2011).
35. T. Jiang, K. Kindt, D. K. Wu, Transcription factor Emx2 controls stereociliary bundle orientation of sensory hair cells. *eLife* **6**, e23661 (2017).
36. V. Bennett, D. N. Lorenzo, Spectrin- and ankyrin-based membrane domains and the evolution of vertebrates. *Curr. Top. Membr.* **72**, 1–37 (2013).
37. A. Jenny, R. S. Darken, P. A. Wilson, M. Mlodzik, Prickle and Strabismus form a functional complex to generate a correct axis during planar cell polarity signaling. *EMBO J.* **22**, 4409–4420 (2003).
38. D. R. P. Tree, J. M. Shulman, R. Rousset, M. P. Scott, D. Gubb, J. D. Axelrod, Prickle mediates feedback amplification to generate asymmetric planar cell polarity signaling. *Cell* **109**, 371–381 (2002).
39. J.-M. Sobotzik, J. M. Sie, C. Politi, D. D. Turco, V. Bennett, T. Deller, C. Schultz, AnkyrinG is required to maintain axo-dendritic polarity in vivo. *Proc. Natl. Acad. Sci. U.S.A* **106**, 17564–17569 (2009).
40. S. L. Jones, F. Korobova, T. Svitkina, Axon initial segment cytoskeleton comprises a multiprotein submembranous coat containing sparse actin filaments. *J. Cell Biol.* **205**, 67–81 (2014).
41. T. Nagaoka, R. Ohashi, A. Inutsuka, S. Sakai, N. Fujisawa, M. Yokoyama, Y. H. Huang, M. Igarashi, M. Kishi, The Wnt/planar cell polarity pathway component Vangl2 induces synapse formation through direct control of N-cadherin. *Cell Rep.* **6**, 916–927 (2014).
42. A. R. Costa, R. Pinto-Costa, S. C. Sousa, M. M. Sousa, The regulation of axon diameter: From axonal circumferential contractility to activity-dependent axon swelling. *Front. Mol. Neurosci.* **11**, (2018).
43. A. R. Costa, S. C. Sousa, R. Pinto-Costa, J. C. Mateus, C. D. Lopes, A. C. Costa, D. Rosa, D. Machado, L. Pajuelo, B. Y. Rubinstein, I. Mendes Pinto, M. Lampe, P. Aguiar, M. M. Sousa, The membrane periodic skeleton is an actomyosin network that regulates axonal diameter and conduction. *eLife* **9**, e55471 (2020).
44. E. D'Este, D. Kamin, F. Göttfert, A. El-Hady, S. W. Hell, STED nanoscopy reveals the ubiquity of subcortical cytoskeleton periodicity in living neurons. *Cell Rep.* **10**, 1246–1251 (2015).
45. S. F. B. van Beuningen, L. Will, M. Harterink, A. Chazeau, E. Y. van Battum, C. P. Frias, M. A. M. Franker, E. A. Katrukha, R. Stucchi, K. Vocking, A. T. Antunes, L. Slenders, S. Doulkeridou, P. S. Smitt, A. F. M. Altelaar, J. A. Post, A. Akhmanova, R. J. Pasterkamp, L. C. Kapitein, E. de Graaff, C. C. Hoogenraad, TRIM46 controls neuronal polarity and axon specification by driving the formation of parallel microtubule arrays. *Neuron* **88**, 1208–1226 (2015).
46. P. W. Baas, J. S. Deitch, M. M. Black, G. A. Banker, Polarity orientation of microtubules in hippocampal neurons: Uniformity in the axon and nonuniformity in the dendrite. *Proc. Natl. Acad. Sci. U.S.A* **85**, 8335–8339 (1988).
47. P. W. Baas, M. M. Black, G. A. Banker, Changes in microtubule polarity orientation during the development of hippocampal neurons in culture. *J. Cell Biol.* **109**, 3085–3094 (1989).
48. L. C. Kapitein, C. C. Hoogenraad, Building the neuronal microtubule cytoskeleton. *Neuron* **87**, 492–506 (2015).
49. R. P. Tas, A. Chazeau, B. Cloin, M. Lambers, C. C. Hoogenraad, L. C. Kapitein, Differentiation between oppositely oriented microtubules controls polarized neuronal transport. *Neuron* **96**, 1264–1271.e5 (2017).
50. K. W. Yau, P. Schätzle, E. Tortosa, S. Pagès, A. Holtmaat, L. C. Kapitein, C. C. Hoogenraad, Dendrites in vitro and in vivo contain microtubules of opposite polarity and axon formation correlates with uniform plus-end-out microtubule orientation. *J. Neurosci.* **36**, 1071–1085 (2016).
51. D. Sun, C. L. Leung, R. K. Liem, Characterization of the microtubule binding domain of microtubule actin crosslinking factor (MACF): Identification of a novel group of microtubule associated proteins. *J. Cell Sci.* **114**, 161–172 (2001).
52. D. Zhou, S. Lambert, P. L. Malen, S. Carpenter, L. M. Boland, V. Bennett, AnkyrinG is required for clustering of voltage-gated Na channels at axon initial segments and for normal action potential firing. *J. Cell Biol.* **143**, 1295–1304 (1998).
53. G. Das, A. Jenny, T. J. Klein, S. Eaton, M. Mlodzik, Diego interacts with Prickle and Strabismus/Van Gogh to localize planar cell polarity complexes. *Development* **131**, 4467–4476 (2004).
54. K. R. Smith, K. J. Kopeikina, J. M. Fawcett-Patel, K. Leaderbrand, R. Gao, B. Schürmann, K. Myczek, J. Radulovic, G. T. Swanson, P. Penzes, Psychiatric risk factor ANK3/Ankyrin-G nanodomains regulate the structure and function of glutamatergic synapses. *Neuron* **84**, 399–415 (2014).
55. J. W. Hammond, C. F. Huang, S. Kaech, C. Jacobson, G. Banker, K. J. Verhey, Posttranslational modifications of tubulin and the polarized transport of kinesin-1 in neurons. *Mol. Biol. Cell* **21**, 572–583 (2010).
56. H. Tao, M. Suzuki, H. Kiyonari, T. Abe, T. Sasaoka, N. Ueno, Mouse prickle1, the homolog of a PCP gene, is essential for epiblast apical-basal polarity. *Proc. Natl. Acad. Sci. U.S.A* **106**, 14426–14431 (2009).

57. S. N. Ehaideb, A. Iyengar, A. Ueda, G. J. Iacobucci, C. Cranston, A. G. Bassuk, D. Gubb, J. D. Axelrod, S. Gunawardena, C.-F. Wu, J. R. Manak, Prickle modulates microtubule polarity and axonal transport to ameliorate seizures in flies. *Proc. Natl. Acad. Sci. U.S.A* **111**, 11187–11192 (2014).
58. J. Olofsson, K. A. Sharp, M. Matis, B. Cho, J. D. Axelrod, Prickle/spiny-legs isoforms control the polarity of the apical microtubule network in planar cell polarity. *Development* **141**, 2866–2874 (2014).
59. C. Liu, C. Lin, D. T. Whitaker, H. Bakeri, O. V. Bulgakov, P. Liu, J. Lei, L. Dong, T. Li, A. Swaroop, Prickle1 is expressed in distinct cell populations of the central nervous system and contributes to neuronal morphogenesis. *Hum. Mol. Genet.* **22**, 2234–2246 (2013).
60. M. I. H. Chowdhury, T. Nishioka, N. Mishima, T. Ohtsuka, K. Kaibuchi, D. Tsuboi, Prickle2 and Igsf9b coordinately regulate the cytoarchitecture of the axon initial segment. *Cell Struct. Funct.* **45**, 143–154 (2020).
61. S. J. Warrington, H. Strutt, K. H. Fisher, D. Strutt, A dual function for Prickle in regulating Frizzled stability during feedback-dependent amplification of planar polarity. *Curr. Biol.* **27**, 2784–2797.e3 (2017).
62. M. Harterink, K. Vocking, X. Pan, E. M. Soriano Jerez, L. Slenders, A. Fréal, R. P. Tas, W. J. van de Wetering, K. Timmer, J. Motshagen, S. F. B. van Beuningen, L. C. Kapitein, W. J. C. Geerts, J. A. Post, C. C. Hoogenraad, TRIM46 organizes microtubule fasciculation in the axon initial segment. *J. Neurosci.* **39**, 4864–4873 (2019).
63. A. Abouelezz, H. Stefen, M. Segerstråle, D. Micinski, R. Minkeviciene, L. Lahti, E. C. Hardeman, P. W. Gunning, C. C. Hoogenraad, T. Taira, T. Fath, P. Hotulainen, Tropomyosin Tpm3.1 is required to maintain the structure and function of the axon initial segment. *iScience* **23**, 101053 (2020).
64. G. Zhong, J. He, R. Zhou, D. Lorenzo, H. P. Babcock, V. Bennett, X. Zhuang, Developmental mechanism of the periodic membrane skeleton in axons. *eLife* **3**, e04581 (2014).
65. N. Unsain, M. D. Bordenave, G. F. Martinez, S. Jalil, C. von Bilderling, F. M. Barabas, L. A. Masullo, A. D. Johnstone, P. A. Barker, M. Bisbal, F. D. Stefani, A. O. Cáceres, Remodeling of the actin/spectrin membrane-associated periodic skeleton, growth cone collapse and F-actin decrease during axonal degeneration. *Sci. Rep.* **8**, 3007 (2018).
66. Y. Qu, I. Hahn, S. E. D. Webb, S. P. Pearce, A. Prokop, Periodic actin structures in neuronal axons are required to maintain microtubules. *Mol. Biol. Cell* **28**, 296–308 (2017).
67. C. O. Brown, J. Uy, N. Murtaza, E. Rosa, A. Alfonso, S. Xing, B. M. Dave, S. Kilpatrick, A. A. Cheng, S. H. White, J. Howe, S. W. Scherer, Y. Lu, K. K. Singh, Disruption of the autism-associated gene SCN2A alters synaptic development and neuronal signaling in patient iPSC-glutamatergic neurons. bioRxiv 2021.09.14.460368 [Preprint]. 16 September 2021. https://doi.org/10.1101/2021.09.14.460368.
68. Z. Que, M. I. Olivero-Acosta, J. Zhang, M. Eaton, W. C. Skarnes, Y. Yang, Sodium channel Nav1.2-L1342P variant displaying complex biophysical properties renders hyperexcitability of cortical neurons derived from human iPSCs. bioRxiv 2021.01.18.427192 [Preprint]. 5 February 2021. https://doi.org/10.1101/2021.01.18.427192.
69. S. Honnappa, S. M. Gouveia, A. Weisbrich, F. F. Damberger, N. S. Bhavesh, H. Jawhari, I. Grigoriev, F. J. van Rijssel, R. M. Buey, A. Lawera, I. Jelesarov, F. K. Winkler, K. Wüthrich, A. Akhmanova, M. O. Steinmetz, An EB1-binding motif acts as a microtubule tip localization signal. *Cell* **138**, 366–376 (2009).
70. M. T. Butler, J. B. Wallingford, Control of vertebrate core planar cell polarity protein localization and dynamics by Prickle 2. *Development* **142**, 3429–3439 (2015).
71. N. P. Andrews, J. X. Boeckman, C. F. Manning, J. T. Nguyen, H. Bechtold, C. Dumitras, B. Gong, K. Nguyen, D. van der List, K. D. Murray, J. Engebrecht, J. S. Trimmer, A toolbox of IgG subclass-switched recombinant monoclonal antibodies for enhanced multiplex immunolabeling of brain. *eLife* **8**, e43322 (2019).
72. N. H. Piguel, S. Fievre, J. M. Blanc, M. Carta, M. M. Moreau, E. Moutin, V. L. Pinheiro, C. Medina, J. Ezan, L. Lasvaux, F. Loll, C. M. Durand, K. Chang, R. S. Petralia, R. J. Wenthold, F. A. Stephenson, L. Vuillard, H. Darbon, J. Perroy, C. Mulle, M. Montcouquiol, C. Racca, N. Sans, Scribble1/AP2 complex coordinates NMDA receptor endocytic recycling. *Cell Rep.* **9**, 712–727 (2014).

Acknowledgments: We thank D. Wu (NIDCD/NIH, USA) for supplying Prickle2 antibody, V. Bennett (Duke Cancer Institute, USA) for supplying the AnkG270-GFP and AnkG190-GFP constructs, C. Hoogenraad (Utrecht University, The Netherlands) for the AnkG480-GFP cDNA, and M. Deans (University of Utah, USA) for GFP-Prickle2 cDNA construct. We thank C. Medina, A. Barranger, I. Seynat, S. Dos Santos Carvalho, J. Stanic, and C. Lefranc for technical assistance. We thank the personnel of the Animal Facility of the Neurocentre Magendie for the rat care. We thank the Bordeaux Neurocampus core facilities used in this study and supported by the LabEx BRAIN (grant ANR-10-LABX-43), including the Biochemistry facility BioProt, and the assistance of Y. Rufin for GST production and purification is acknowledged. We thank E. Pacary for initial advices of IUE in mice. Confocal and STORM imaging were done at the Bordeaux Imaging Center, a service unit of the CNRS-INSERM and Bordeaux University, member of the national infrastructure France BioImaging supported by the French National Research Agency (ANR-10-INBS-04). The help of F. Cordelières for the AIS macro for ImageJ and M. Mondin for STORM imaging is acknowledged. All of the above facilities were funded by the LabEx BRAIN (ANR-10-LABX-43). We thank M. Grubb and M. Engelhardt for supportive and constructive comments and C. Leterrier for advices on actin staining and imaging on STORM. **Funding:** This work was supported by Institut National de la Santé et de la Recherche Médicale INSERM (to M.M. and N.S.), Fondation pour la Recherche Médicale (FRM) "Equipe FRM 2016" DEQ20160334899 (to M.M.), Ministère de l'Enseignement Supérieur et de la Recherche (MESRI) (to A.D.-R.), LabEx BRAIN ANR-10-LABX-43 (to A.D.-R.), Bordeaux Neurocampus Startup grant by the Region Aquitaine 2018.599 (to A.B.), and IDEX ATTRACTIVITE Chaires Neurocampus of the University of Bordeaux (to A.B.). The Planar Polarity Team is part of the GPR Brain_2030 Nanocoding project. **Author contributions:** Conceptualization: M.M., N.S., and A.D.-R. Methodology: M.M., N.S., and A.D.-R. Investigation: A.D.-R., M.M.M., J.E., M.M., S.P.-C., N.A., J.D.N., C.B., A.F., A.B., N.S., and F.C. Supervision: M.M., J.E., and N.S. Writing—original draft: M.M. and A.D.-R. Writing—review and editing: A.D.-R., M.M., J.E., A.B., and N.S. **Competing interests:** The authors declare that they have no competing interests. **Data and materials availability:** All data needed to evaluate the conclusions in the paper are present in the paper and/or the Supplementary Materials.

Submitted 17 February 2022
Accepted 25 July 2022
Published 9 September 2022
10.1126/sciadv.abo6333
