# Supplementary material for: The core PCP protein Prickle2 regulates axon number and AIS maturation by binding to AnkG and modulating microtubule bundling
Source: Sci Adv. 2022 Sep 9;8(36):eabo6333. doi: 10.1126/sciadv.abo6333 (PMC9462691; doi:10.1126/sciadv.abo6333)
Supplement: Supplementary file 2 — Figs. S1 to S6 [file sciadv.abo6333_sm.v2.pdf]

CORRECTED 7 MAY 2024

Science Advances
AAAS

Supplementary Materials for

The core PCP protein Prickle2 regulates axon number and AIS maturation by binding to AnkG and modulating microtubule bundling

Ana Dorrego-Rivas *et al.*

Corresponding authors: Ana Dorrego-Rivas, ana.dorrego-rivas@kcl.ac.uk; Nathalie Sans, nathalie.sans@inserm.fr

Sci. Adv. **8**, eabo6333 (2022)
DOI: 10.1126/sciadv.abo6333

This PDF file includes:

Figs. S1 to S6

Figure S1 (related to Figure 1)

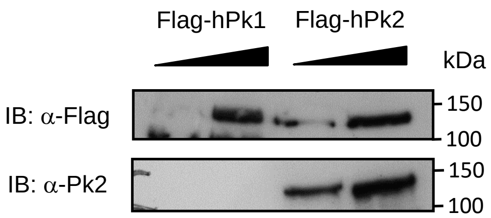

Fig. S1. Characterization of Pk2 antibody specificity. Anti-Pk2 detects a band in between 100 and 150 kDa, corresponding to Pk2 molecular weight in Flag-Pk2 overexpressing cells, but it does not recognize Flag-Pk1. Anti-flag was used as a control.

Figure S2 (related to Figure 4)

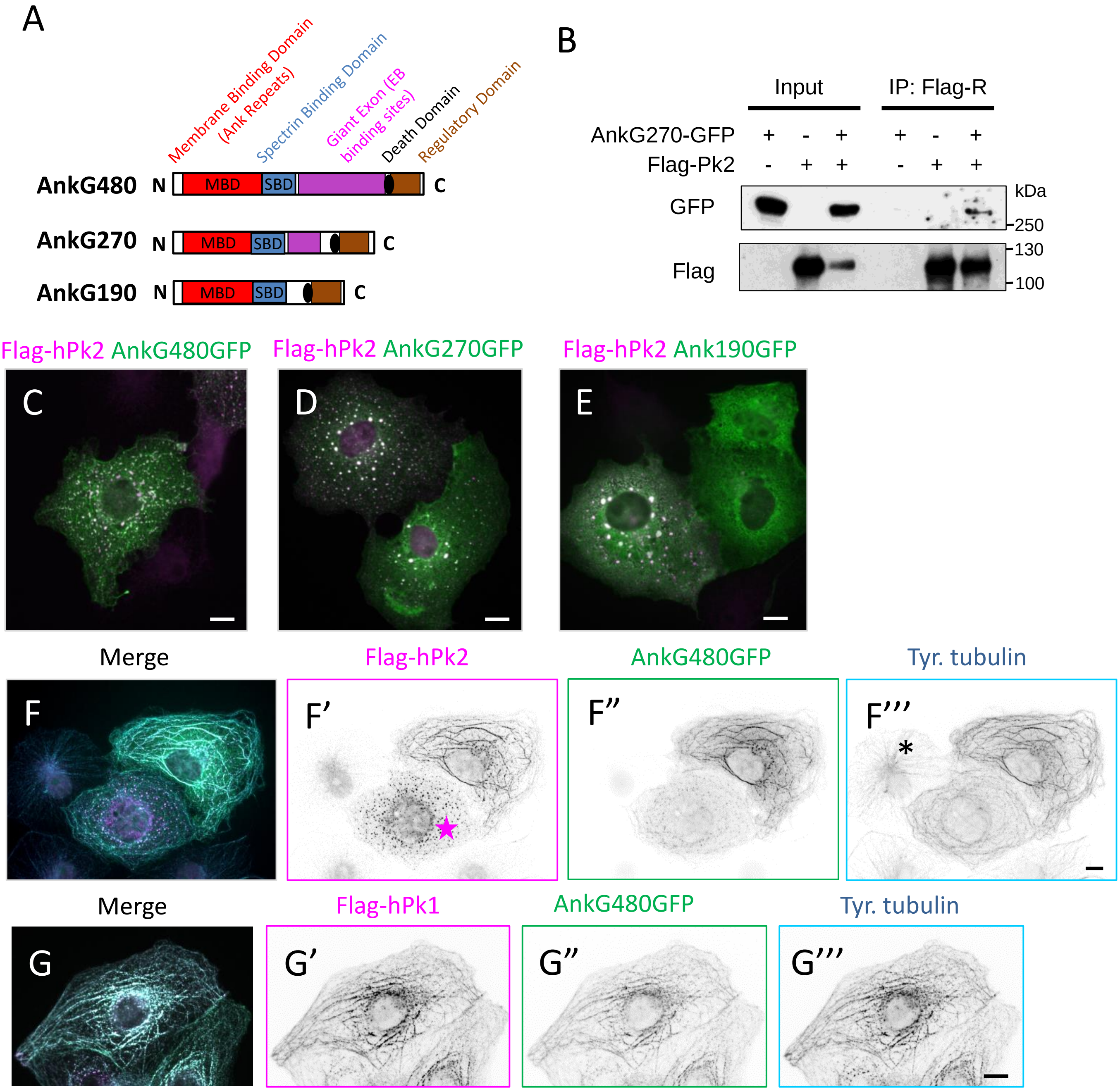

Fig. S2. Pk2 and Pk1 bundling activities. (**A**) Domain organization of neuronal ankyrins. The membrane-binding domain (MBD, red) comprised of 24 ankyrin repeats and the spectrin-binding domain (SBD) are common to all three isoforms. The giant ankyrin isoforms (AnkG270 & 480) have an insertion of a single exon (magenta), comprised of EB binding sites. Other functional domains include a death domain (black oval) and a C-terminal unstructured regulatory domain (brown). (**B**) AnkG270-GFP co-immunoprecipates with hPk2-flag *in vitro*. (**C-E**). Illustration of COS-7 transfected with Flag-Pk2 and the three isoforms of AnkG colocalizing in clusters/aggregates. (**F-F'''**) Illustration of a circular MT-bundling phenotype in a COS-7 cell transfected with Flag-Pk2 and AnkG480-GFP. Note the cell in F' (star) with weaker levels of AnkG and the cell in F''' (asterisk) with no transfection that have no MT-bundling. (**G-G'''**). The co-expression of Flag-Pk1 with AnkG480-GFP promotes the formation of microtubule bundles. A total of 213 cells were quantified from one experiment. Scalebars: 10 μm.

Figure S3 (related to Figure 5)

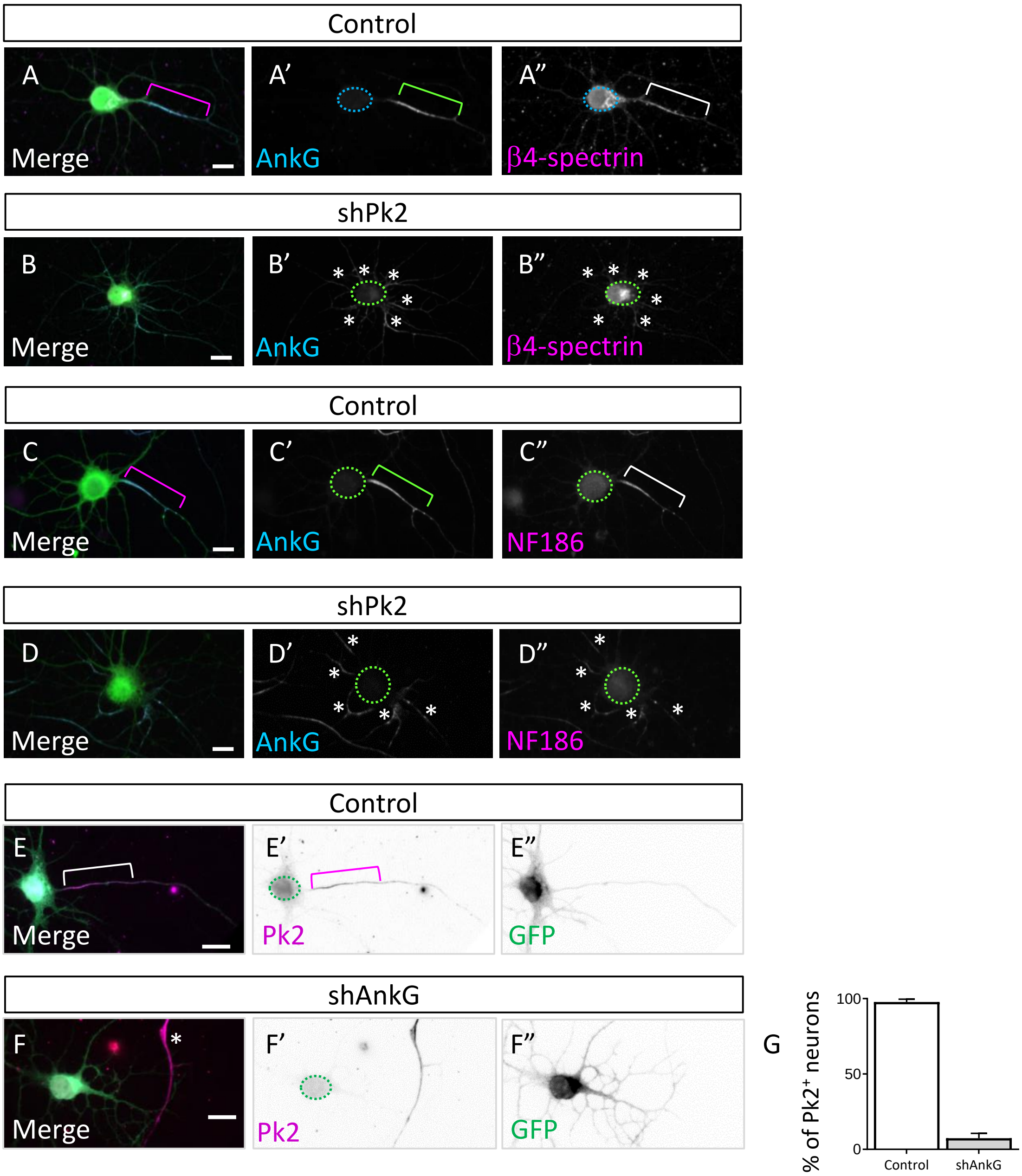

Fig. S3. Pk2 regulates AIS formation and its presence at the AIS is AnkG-dependent. (**A-D''**) Illustration of neurons depleted for Pk2 and labelled at DIV7 for AIS markers β4 spectrin and NF186. Scalebars: 10 µm. Bracket indicate AIS. Asterisks indicate AnkG-positive neurites. (**E-F''**) Neurons depleted for AnkG have almost no Pk2 staining. Asterisk in F indicates the axon of a non transfected neuron with strong AnkG labelling. Dotted circle indicates the soma. (**G**) Percentage of Pk2/AnkG-positive neurons in control and shAnkG cells. Data are show as mean ± SEM. N= 98 neurons for control and n=125 for shAnkG, from 3 independent experiments. Scalebars: 10 µm.

Figure S4 (related to Figure 6)

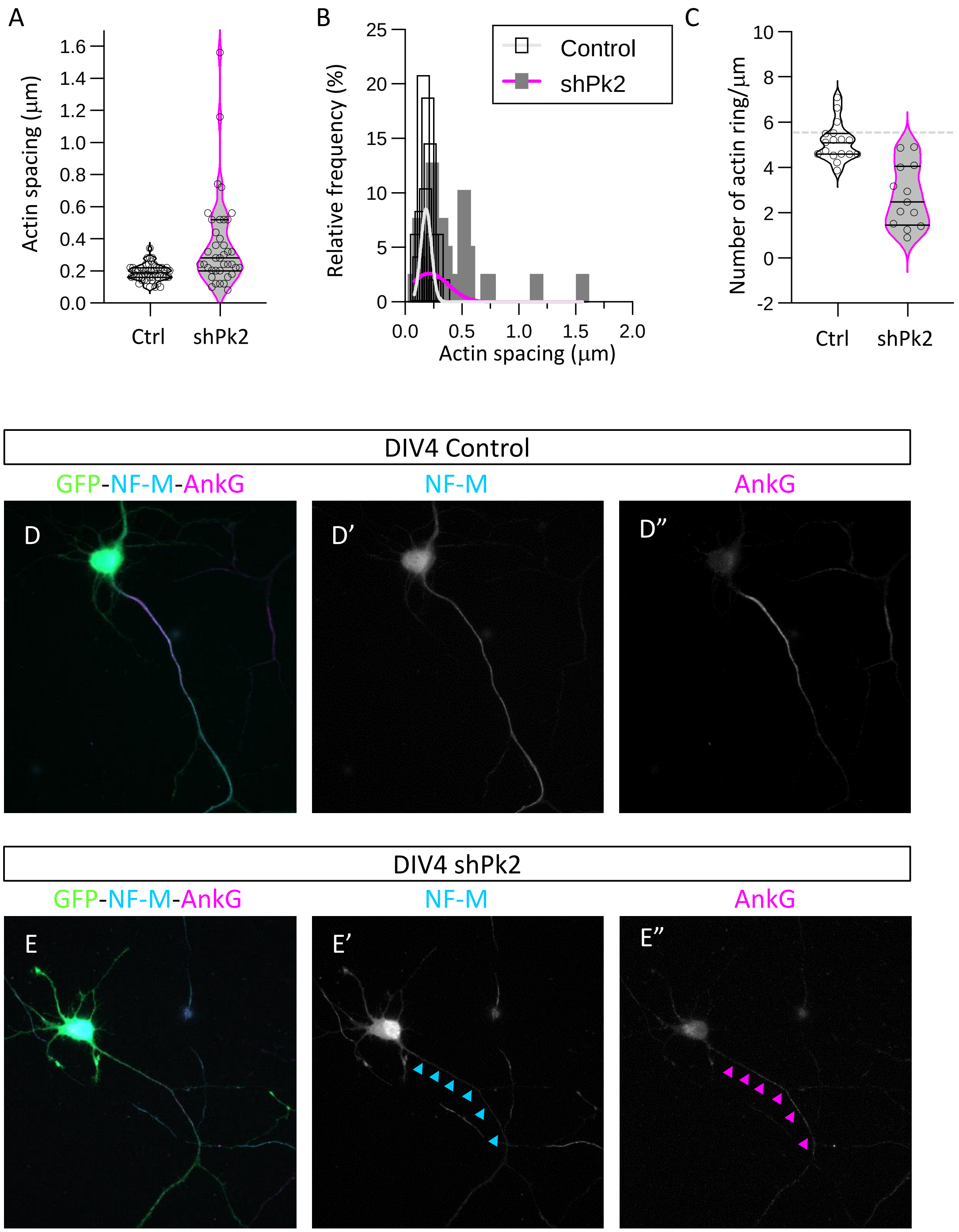

Fig. S4. Pk2 regulates cytoskeleton organization. (**A-C**) Quantifications of the disruption of the actin periodicity at the AIS. The spacing between two actin rings is increased (A) while their overall number is severely reduced (B,C). (**D-E''**) Neurons depleted for Pk2 with a fragmented and reduced AnkG labeling exhibit a strong decrease in neurofilament (NF-M) expression levels. Data are show as mean ± SEM. n= 48 from 3 neurons for control and n=39 from 3 neurons for shPk2 from 2 independent experiments. Scalebars: 10 µm.

Figure S5 (related to Figure 7)

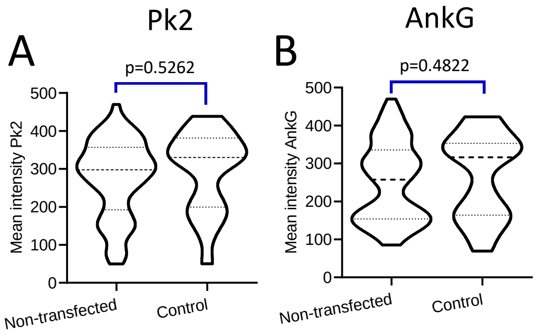

Fig. S5. A control shRNA does not affect endogenous Pk2 or AnkG levels *in vivo*. (**A-B**) Quantifications of fluorescence intensity levels of Pk2 (A) or AnkG (B) in shControl neurons compared to surrounded and non-transfected neurons. For Pk2 p=0.5262 from Welch's t-test, for AnkG P=0.4822 from Mann Whitney's test, N=3 brains, n=20 shControl neurons and n=75 surrounding cells.

Figure S6

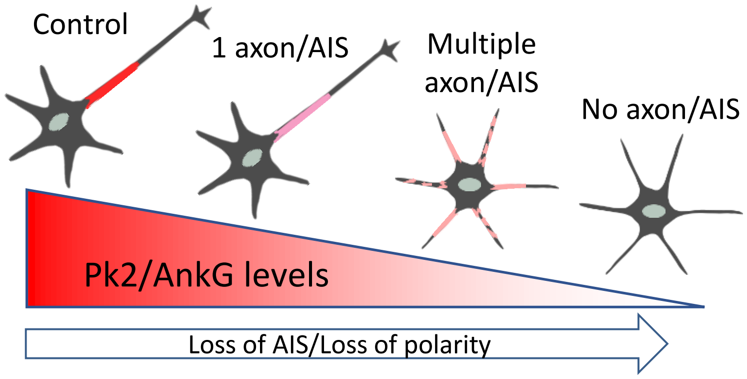

Fig. S6. Prickle2 and AnkG are co-dependent during neuronal polarity and AIS formation. Prickle2 (Pk2) depletion correlates with AnkyrinG (AnkG) levels decreased and with an increasing loss of polarity. A small depletion of Prickle2 does not affect neuronal polarity (1 axon), but affects the AIS integrity with reduced AnkG levels and sometimes fragmented AIS. Reducing Pk2 levels more alters both neuronal polarity as illustrated by a multiple axon phenotype, and the AIS, with reduced AnkG levels and fragmented AIS. When Pk2 depletion is severe, there is a complete loss of polarity (no axon/AIS).
